# Supplementary material for: Nascent craft specialization in the Pre-Pottery Neolithic A? Bead making at Shubayqa 6 (northeast Jordan)
Source: PLoS One. 2023 Dec 8;18(12):e0292954. doi: 10.1371/journal.pone.0292954 (PMC10707568; doi:10.1371/journal.pone.0292954)
Supplement: S2 Appendix — (PDF) [file pone.0292954.s002.pdf]

S2 Appendix - Greenstone bead experiment report

Introduction

Among the bead types recorded at Shubayqa 6, circular disc beads made from the so-called "Dabba Marble" green variety are predominant. Evidence of on-site production covering the entire *chaîne opératoire* of this type has been found too. The major aim of this experimental program is to understand the production sequence of the disc beads and the technical investment required for their manufacture. More precisely, it aims at:

1) Corroborating experimentally the production process that have resulted into five successive states of artefacts observed archaeologically: Primary blank -> preforms -> perforated preforms -> shaped/polished beads -> final products (finished beads). Therefore, the experimental work was divided into four main production stages, similar to those experiments conducted by other authors (see for example Wright et al. [41], Bains [57] and Gurova & Bonsall [78]), which involved reducing the raw material, then shaping the preforms, drilling of the roughout, and the final polish.
2) Testing the appropriateness of specific techniques applied for each stage, the efficiency of tools and the approximate time needed to achieve each stage.

The experimental protocol

Four successive stages were experimented, which resulted in the production of 8 beads (S1 Table 1) and 5 roughouts (S1 Table 2). About 4 beads and 2 roughouts were furthermore produced, but their production was not timed and therefore not present in the appended tables. The raw materials used for the experiment consisted of a piece of green Dabba Marble (see S1 Fig 1). This had been retrieved from Wadi Jilat, which is considered one of the potential sources for the ancient beads that were produced at Shabyqa 6.

Replica flint blades and drills were used for the production stages that involved reducing and perforating the preforms. For the shaping of the preforms and the final polishing, replica handstones of vesicular and non-vesicular basalt were used. These were all provided by Johan Villemoes and based on tools found at Shubayqa 6, which also appear frequently in EPPNA and LPPNA contexts. Sinew from a recently slaughtered goat was used to string the beads in the final polishing stage, and a piece of leather was used for stabilising the bead during drilling. As Auxiliar agents, abrasive were occasionally used in the form of local, middle sized grained sand as well as water during the abrasion and drilling process.

Production Stages	Bead 1	Bead 2	Bead 3	Bead 4	Bead 5	Bead 6	Bead 7	Bead 8
**Reducing**	11:50 min.	11:50 min.	-	-	-	11:30 Min.	11:30 min.	20 min.
**Initial abrasion**	30 min.	30 min.	8:30 min.	14:30 min.	15:30 min.	12:00 min.	12:00 min.	8 min.
**Cutting**	2:15 min.	2:15 min.	n/a	2:30 min.	2:30 min.	2:15 min.	2:15 min.	n/a
**Drilling**	10:30 min.	-	16:11 min.	5:52 min.	2 min.	7:40 min.	6:30 min	40 min.
**Final shaping**	11:17 min	-	6:30 min.	-	3:20 min	-	8:03 min	30 min.
**Polishing**	n/a	21:45 min	21:45 min	21:45 min	21:45 min	21:45 min	n/a	n/a

S2 Table 1. Time measures for all the finished beads according to the different manufacturing stages.

Failed roughouts	Roughout 1	Roughout 2	Roughout 3	Roughout 4	Roughout 5
**Breakage stage**	Perforation	Perforation	Perforation	Perforation	Final polishing
**Cause of breakage**	Too much pressure + misaligned perforation	Perforation was off centre, which caused the edge to break	Perforation misaligned	Tried to widen uniconically drilled perforation	Perforation was off-centre and too near the edge. The roughout thereby broke while being grinded with the other beads

S2 Table 2. Overview of the failed roughouts and the cause of breakage.

Reducing the raw material

At first Johan Villemoes tried to reduce a piece of greenstone (Dabba marble) retrieved from Wadi Jilat into bead preforms by knapping the raw material with a hammer stone made from sandstone. However, it turned out to be difficult to control how the greenstone was flaking by knapping only. Therefore, given the small sample of greenstone provided for this experiment, it was decided to quit this approach in order to save as much of the fragment as possible. Instead, we decided to cut some grooves in the greenstone in order to improve control of the flaking. Wedging the greenstone after cutting and then knap it turned out to be the most efficient approach. It took approximately 10-20 minutes to produce flakes useful for bead manufacturing. During the knapping tiny fragments of greenstone were created, similar to the raw material debris found at Shubayqa 6.

S2 Fig 1. The piece of green Dabba Marble from Wadi Jilat that was used for the experiment.

Abrading the reduced products into preforms

The next stage involved shaping the flakes into rectangular preforms, similar to those observed archaeologically. This was done by grinding the preforms in reciprocal motions against basalt (see S1 Fig 2). Both vesicular and non-vesicular basalts were used for this, but there seemed to be no apparent macroscopic difference between the final products.

It took about 8-30 minutes to do this (see table 1), depending on the size of the worked greenstone. By adding water and sand to the process, the grinding efficiency increased and the surface of the preform became smoother.

S2 Fig 2. Abrading the greenstone fragment into a rectangular preform on non-vesicular basalt.

Cutting the preforms

In some cases the preforms were cut into smaller pieces in order to create multiple roughouts (see S1 Fig 3). Abrading large pieces and then cutting them into smaller roughouts resulted, indeed, in an easier task than abrading tiny pieces. The cutting was done by sawing the greenstone with a flint blade. The flint blade could easily cut through the preform and once a groove had been made halfway through the preform, one could almost effortlessly snap the greenstone. One roughout with this kind of grooved mark was found among the Shubayqa 6 assemblage, making it plausible that this was a technique also used by the bead makers on the site.

As shown in S1 Table 1, this process usually took less than 3 minutes to conclude, depending on the size of the preform and when water was added, the cutting speed further increased.

S2 Fig 3. Cutting the greenstone with a flint blade

Drilling the preforms

About half of the roughouts were drilled manually, meaning through rotation of a flint drill by one hand (Fig. 4), while the others were mechanically drilled by using a shaft mounted hand drill produced by A. Ruter, which could be rotated between the hands (see S1 Table 3). For the first technique, it was easy to stabilise the roughouts while drilling despite their small sizes. This could be done by either holding the roughout on a flat surface and steady it with the hand or by just keeping it in one hand, while drilling with the other. When using the mechanical drill it was necessary to use both hands, but the roughout could be steadied by placing the roughout on a piece of leather. It usually took between 6-15 minutes to drill by hand and 5-16 minutes to drill it mechanically. Drilling by hand was more exhausting, which meant that although there was no apparent time difference between the two methods, more roughouts could be produced with less effort when the mechanical drill was used.

Drilling technique	Bead 1	Bead 2	Bead 3	Bead 4	Bead 5	Bead 6	Bead 7	Bead 8
Hand drilled	X	X			X		X	X
Mechanically drilled		X	X	X	X	X		

S2 Table 3. The used drilling techniques for the finished beads.

The majority of the beads were drilled biconically. Two roughouts were uniconically drilled (Bead 8 + Roughout 4) which turned out to be a slower process. It took 40 minutes to drill Bead 8 this way and Roughout 4 broke while trying to extend the perforation from the other side. When drilling from one side only, the perforation hole also tended to get wider rather than deeper.

Some of the roughouts broke during the drilling process, either due to an off-centred or misaligned perforation or because too much pressure was applied during the final stage of drilling. It turned out that rectangular roughouts were best suited for drilling, as one could centre the perforation more easily.

Two roughouts of the same size were hand drilled biconically with and without water and sand as abrasives.
Roughout without abrasives - 15:01 min
Roughout drilled with abrasives – 13:02 min

This shows that abrasives helps to a small extent in decreasing the time spent on drilling. Microscopic analysis can further help indicate whether the perforation marks left by the various methods will be different.

S2 Fig 4. Drilling of rectangular roughout.

Shaping the final roughouts

The drilled roughouts were then once again abraded individually on a basalt surface in order to reproduce the characteristic disc shape. This stage was executed in the same way as the initial abrasion stage, but took less time (usually about 3-11 minutes). The time difference must account for various sizes and thickness of the roughouts.

Polishing

The final polishing of the beads was conducted by stringing 6 nearly finished beads together and then grinding them on a slab of basalt. The first attempt failed after 8 minutes because the beads were not strung together tightly enough and the string also broke. It was further complicated by the fact that the surface was not flat enough, which meant that the beads were not ground evenly.

It was then decided to use sinew from goat (see S1 Fig 5). The sinew were leftovers from an earlier experiment) to string the beads on a new flat basalt surface. The sinew proved stronger and more useful than the modern string, and following further 13 minutes and 45 seconds of grinding (21 minutes and 45 seconds in total), the experiment was concluded. The beads had reached the desired sharp edges and were further reduced to the same size.

In hindsight and based on other studies using this technique, the experiment would probably have been more efficient if more beads were added to the string, as it took a lot of effort to get a good grip on the beads and to keep them tight together (See S1 Fig 6).

S2 Fig 5. The beads strung together with sinew.

S2 Fig 6. ***En masse*** **polishing.**

Conclusion

The objectives of this experiment was achieved, as we managed to successfully reproduce beads that are similar to the greenstone disc beads that have been excavated from Shubayqa 6 through the proposed production sequence (see S1 Fig 7). It was furthermore possible to identify techniques that increased the efficiency of each production stage, such as the cutting of grooves to provide rectangular routhouts that eventually made the centre of drilling easier, the biconically drilling for preventing breakage, and the use of hand shafted tools that was less exhausting than drilling by hand. Each stage was timed, indicating that the whole work process behind the making of one bead could take up to 45 to 66 minutes for an unskilled person. Given the high density of beads at Shubayqa 6 it must however be assumed that these bead makers had achieved higher proficiency and could do this process faster. About 5 out of 13 preforms broke during the production, which also fits the high amount of failed roughouts in the stone bead assemblage from the site. These mostly broke during the perforation stage that seems to have been the most critical point during manufacture and which required most attention. Furthermore, the experiment produced greenstone debris that resembles the greenstone raw material recovered during excavation. Although the suggested production sequence have been proven to work, it was noted that this sequence could not be directly applied to the production of other beads made from different raw material, as the appropriate techniques depend on the properties of the given mineral. This was demonstrated with our other experiments on calcite and ostrich eggshells beads.

S2 Fig 7. A selection of the beads that were produced during this experiment.
One roughout that broke during drilling and two nearly finished beads can be seen in the upper register, while the lower register contains the six beads that were used for the final polishing experiment, including the one that broke.

References

78. Gurova M, Bonsall C. Experimental Replication of Stone, Bone and Shell Beads from Early Neolithic Sites in Southeast Europe. In: Mayer DEB, Bonsall C, Choyke AM, editors. Not Just for Show: The Archaeology of Beads, Beadwork, and Personal Ornament. Havertown: Oxbow Books; 2017. pp.159-167.
