# Supplementary material for: Nascent craft specialization in the Pre-Pottery Neolithic A? Bead making at Shubayqa 6 (northeast Jordan)
Source: PLoS One. 2023 Dec 8;18(12):e0292954. doi: 10.1371/journal.pone.0292954 (PMC10707568; doi:10.1371/journal.pone.0292954)
Supplement: S4 Appendix — (PDF) [file pone.0292954.s004.pdf]

Certificate of Calibration

XL2 PMA QC Sheet
Document #: FRM 123
Revision: K
Revision Date: tbd

Serial Number:	87920	Software:	8.4I.10	Date of Q.C.:	18-May-17
Resolution:	138,64	Tube	Ag 38kV	Inspector:	TB

30 second analysis time

Pure Au (avg of 3)

	Expected	Low	High	Measured	Err	
Ag		0	0,07	0,00	0,01	<LOD
Pd		0	0,07	0,00	0,00	<LOD
Rh		0	0,07	0,00	0,01	<LOD
Ru		0	0,07	0,00	0,01	<LOD
Au	100	99,7	100,0	99,94	0,21	OK
Pt		0	0,07	0,00	0,06	<LOD
Ir		0	0,07	0,05	0,03	<LOD
Zn		0	0,07	0,00	0,03	<LOD
Cu		0	0,07	0,00	0,01	<LOD
Ni		0	0,07	0,00	0,02	<LOD
Co		0	0,07	0,00	0,02	<LOD
Fe		0	0,07	0,02	0,03	<LOD
Karat	14	23,9	24,0	23,98		OK

Pure Pt (avg of 3)

	Expected	Low	High	Measured	Err	
Ag		0	0,07	0,01	0,00	<LOD
Pd		0	0,07	0,00	0,00	<LOD
Rh		0	0,07	0,00	0,01	<LOD
Ru		0	0,07	0,00	0,01	<LOD
Au		0	0,07	0,00	0,31	<LOD
Pt	100	99,7	100,0	99,98	0,45	OK
Ir		0	0,07	0,00	0,18	<LOD
Zn		0	0,07	0,00	0,02	<LOD
Cu		0	0,07	0,00	0,05	<LOD
Ni		0	0,07	0,00	0,04	<LOD
Co		0	0,07	0,01	0,02	<LOD
Fe		0	0,07	0,00	0,04	<LOD

Pure Ag (avg of 3)

	Expected	Low	High	Measured	Err	
Ag	100	99,7	100,00	99,99	0,29	OK
Pd		0	0,07	0,00	0,01	<LOD
Rh		0	0,07	0,00	0,01	<LOD
Ru		0	0,07	0,00	0,00	<LOD
Au		0	0,07	0,00	0,02	<LOD
Pt		0	0,07	0,00	0,02	<LOD
Ir		0	0,07	0,00	0,01	<LOD
Zn		0	0,07	0,00	0,01	<LOD
Cu		0	0,07	0,00	0,01	<LOD
Ni		0	0,07	0,00	0,04	<LOD
Co		0	0,07	0,00	0,02	<LOD
Fe		0	0,07	0,00	0,04	<LOD

Pure Cu (avg of 3)

	Expected	Low	High	Measured	Err	
Ag		0	0,07	0,00	0,00	<LOD
Pd		0	0,07	0,00	0,00	<LOD
Rh		0	0,07	0,00	0,00	<LOD
Ru		0	0,07	0,00	0,00	<LOD
Au		0	0,07	0,00	0,02	<LOD
Pt		0	0,07	0,00	0,05	<LOD
Ir		0	0,07	0,04	0,02	OK
Zn		0	0,07	0,03	0,02	<LOD
Cu	100	99,7	100,00	99,98	0,07	OK
Ni		0	0,06	0,00	0,01	<LOD
Co		0	0,07	0,00	0,00	<LOD
Fe		0	0,07	0,00	0,01	<LOD

60 second analysis time

Fluxana 704-16

	Expected	Low	High	Measured	Err	
Ag	3,00	2,85	3,15	2,94	0,03	OK
Pd	12,53	12,33	12,73	12,47	0,07	OK
Rh		0,00	0,05	0,00	0,01	<LOD
Ru		0,00	0,05	0,00	0,01	<LOD
Au	75,07	74,57	75,57	74,88	0,13	OK
Pt		0,00	0,05	0,00	0,04	<LOD
Ir		0,00	0,05	0,00	0,03	<LOD
Zn		0,00	0,05	0,00	0,08	<LOD
Cu	9,40	8,90	9,90	9,73	0,06	OK
Ni		0,00	0,05	0,00	0,02	<LOD
Co		0,00	0,05	0,00	0,01	<LOD
Fe		0,00	0,05	0,00	0,02	<LOD
Karat	18,00	17,75	18,25	17,97		OK

Fluxana 715-16

	Expected	Low	High	Measured	Err	
Ag		0,00	0,10	0,01	0,01	<LOD
Pd		0,00	0,05	0,00	0,00	<LOD
Rh		0,00	0,05	0,00	0,00	<LOD
Ru		0,00	0,05	0,00	0,01	<LOD
Au	58,69	58,19	59,19	58,70	0,12	OK
Pt		0,00	0,05	0,00	0,05	<LOD
Ir		0,00	0,05	0,00	0,02	<LOD
Zn	9,00	8,30	9,80	9,50	0,05	OK
Cu	26,32	25,82	26,82	26,01	0,09	OK
Ni	5,99	5,69	6,29	5,99	0,05	OK
Co		0,00	0,05	0,00	0,01	<LOD
Fe		0,00	0,05	0,00	0,01	<LOD
Karat	14,00	13,75	14,25	14,09		OK

Fluxana 732-16

	Expected	Low	High	Measured	Err	
Ag	58,61	58,1	59,11	58,38	0,15	OK
Pd		0	0,05	0,00	0,01	<LOD
Rh		0	0,05	0,00	0,01	<LOD
Ru		0	0,05	0,00	0,00	<LOD
Au	33,33	32,83	33,83	33,69	0,10	OK
Pt		0	0,05	0,00	0,02	<LOD
Ir		0	0,05	0,00	0,02	<LOD
Zn		0	0,25	0,00	0,02	<LOD
Cu	8,06	7,7	8,46	8,19	0,05	OK
Ni		0	0,05	0,00	0,02	<LOD
Co		0	0,05	0,00	0,01	<LOD
Fe		0	0,05	0,00	0,02	<LOD
Karat	8,00	7,75	8,25	8,08		OK

Fluxana 734-16

	Expected	Low	High	Measured	Err	
Ag	6,2	5,94	6,54	6,29	0,04	OK
Pd		0	0,05	0,00	0,00	<LOD
Rh		0	0,05	0,00	0,00	<LOD
Ru		0	0,05	0,01	0,00	<LOD
Au	33,52	33,02	34,02	33,73	0,11	OK
Pt		0	0,05	0,00	0,04	<LOD
Ir		0	0,05	0,03	0,02	OK
Zn	10,42	9,42	10,92	10,37	0,05	OK
Cu	49,82	48,8	50,80	50,01	0,10	OK
Ni		0	0,05	0,00	0,01	<LOD
Co		0	0,05	0,00	0,01	<LOD
Fe		0	0,05	0,00	0,01	<LOD
Karat	8	7,75	8,25	8,09		OK

Fluxana 743-16

	Expected	Low	High	Measured	Err	
Ag		0,00	0,06	0,00	0,01	<LOD
Pd		0,00	0,06	0,00	0,00	<LOD
Rh		0,00	0,06	0,00	0,00	<LOD
Ru		0,00	0,05	0,00	0,00	<LOD
Au	80,15	79,65	80,65	80,39	0,13	OK
Pt		0,00	0,05	0,00	0,06	<LOD
Ir		0,00	0,05	0,00	0,03	<LOD
Zn	4,27	3,87	4,67	4,26	0,04	OK
Cu	1,05	0,85	1,25	1,06	0,03	OK
Ni	14,53	14,13	14,93	14,32	0,07	OK
Co		0,00	0,05	0,00	0,01	<LOD
Fe		0,00	0,05	0,00	0,03	<LOD
Karat	19,2	18,85	19,45	19,29		OK

Fluxana 744-16

	Expected	Low	High	Measured	Err	
Ag	26,3	25,3	27,7	26,67	0,10	OK
Pd	6,56	6,26	6,86	6,51	0,04	OK
Rh		0	0,05	0,00	0,01	<LOD
Ru		0	0,05	0,00	0,01	<LOD
Au	55,54	55,04	56,04	55,76	0,12	OK
Pt		0	0,05	0,00	0,03	<LOD
Ir		0	0,05	0,00	0,02	<LOD
Zn	1,38	1,08	1,68	1,28	0,03	OK
Cu	10,25	9,25	10,75	9,90	0,06	OK
Ni		0	0,05	0,00	0,02	<LOD
Co		0	0,05	0,00	0,01	<LOD
Fe		0	0,05	0,00	0,02	<LOD
Karat	13,33	13,08	13,6	13,38		OK

Standards used for factory calibrations are certified reference materials (CRM) or reference materials (RM) where available.
Certificates of Analysis are available upon request.

This certificate is issued in accordance with Thermo Fisher Scientific factory specifications.
The measurements were found to be within specification limits at the time of manufacture and calibration.

Thermo Scientific Niton Analyzers | 900 Middlesex Turnpike, Building 8 | Billerica, MA 01821, USA | +1 978-670-7460 | +1 978-670-7430 | www.thermo.com/Niton | 800-875-1578 (toll free)
