# Supplementary figures and images for: Nascent craft specialization in the Pre-Pottery Neolithic A? Bead making at Shubayqa 6 (northeast Jordan)
Source: PLoS One. 2023 Dec 8;18(12):e0292954. doi: 10.1371/journal.pone.0292954 (PMC10707568; doi:10.1371/journal.pone.0292954)

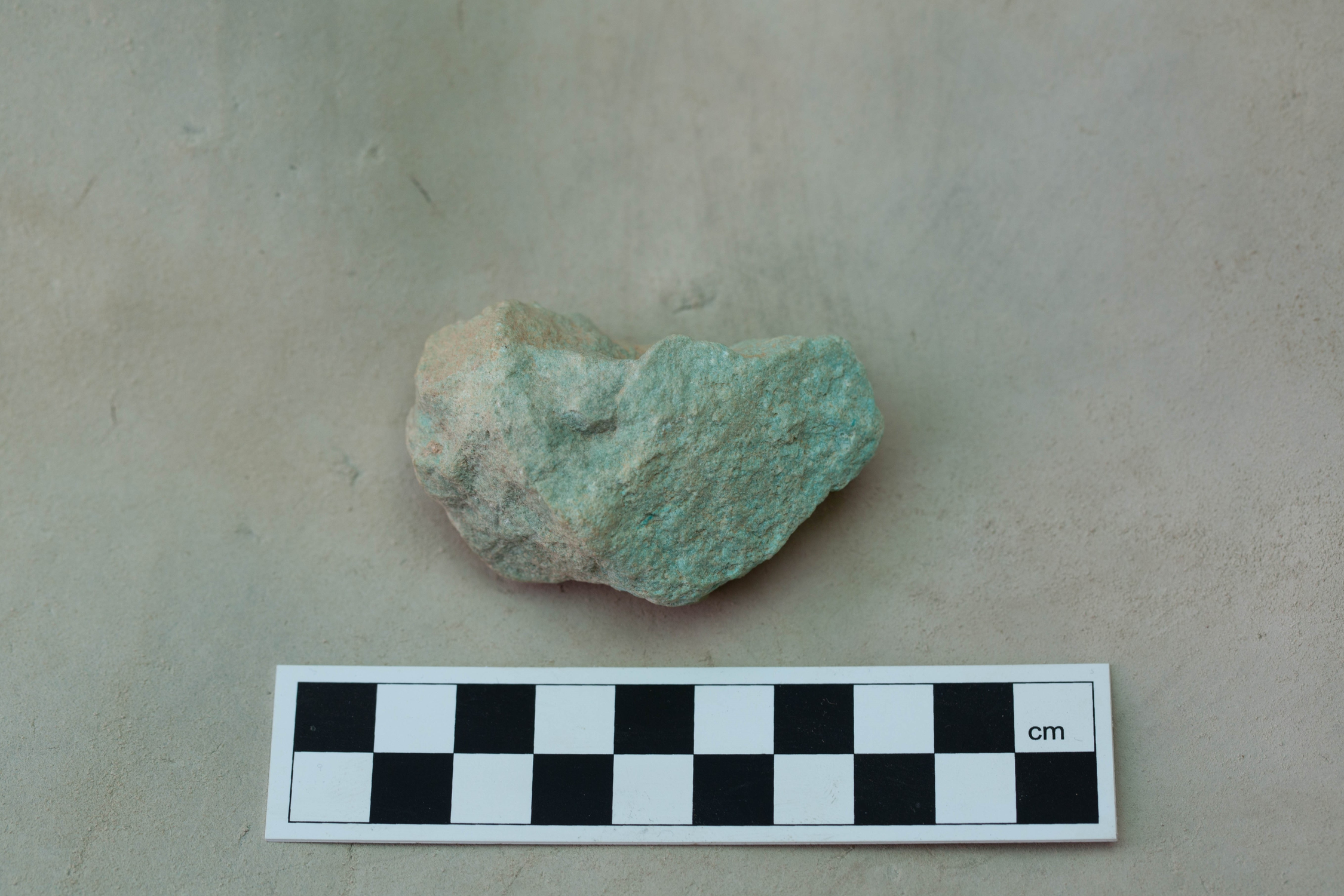

Supplement: S1 Fig — (TIF) [file pone.0292954.s005.tif]

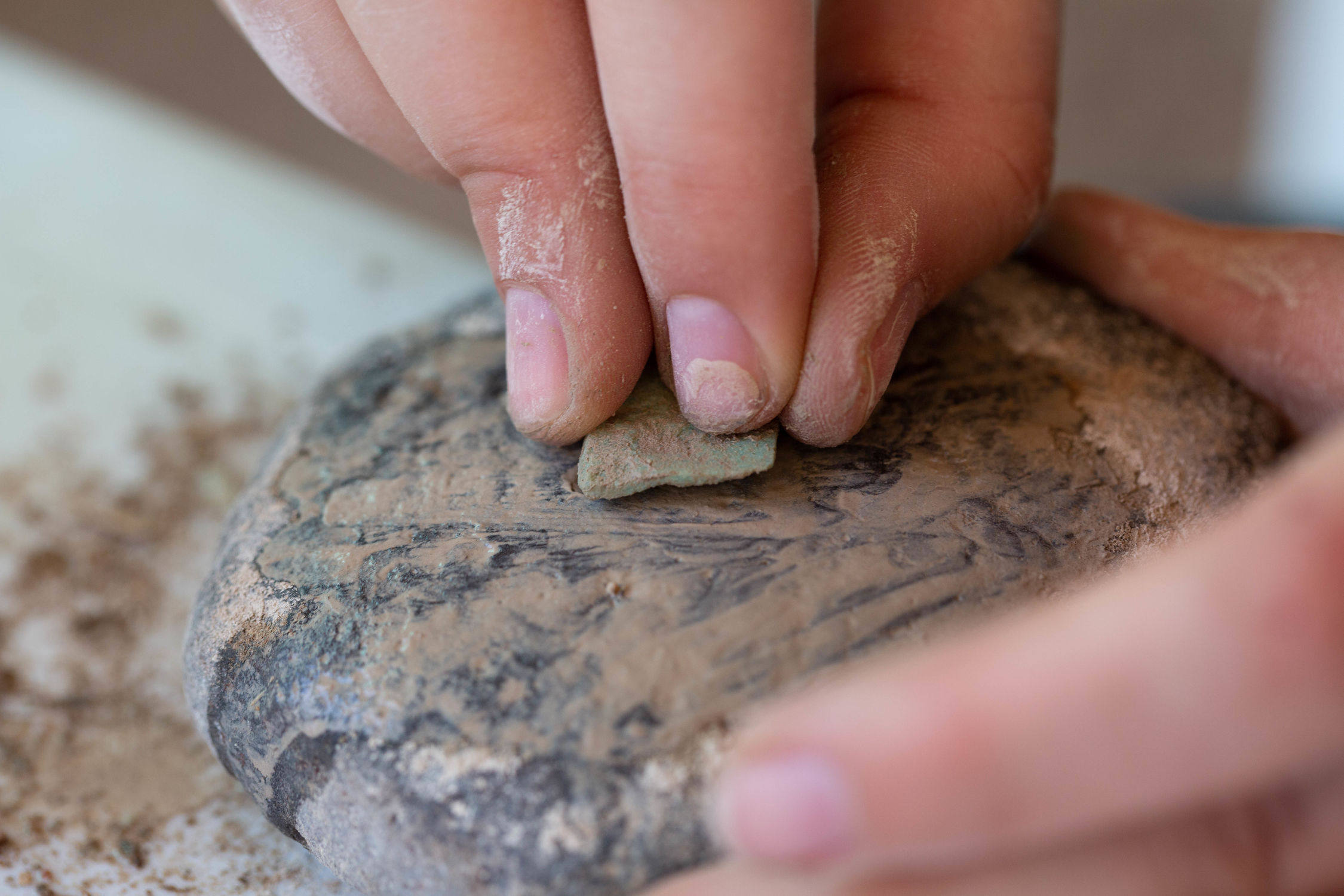

Supplement: S2 Fig — (TIF) [file pone.0292954.s006.tif]

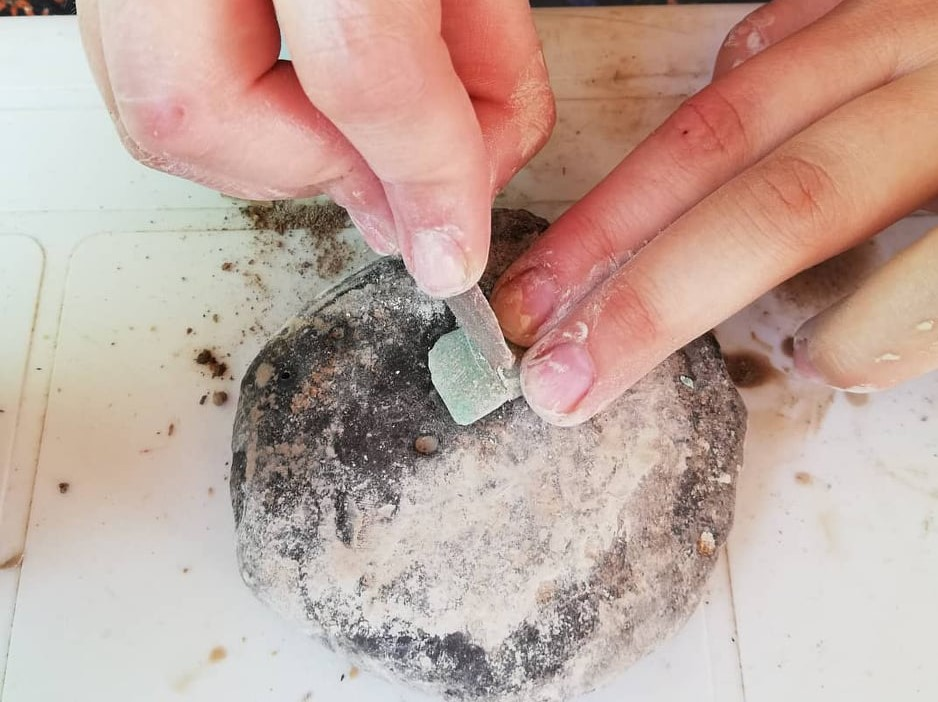

Supplement: S3 Fig — (TIF) [file pone.0292954.s007.tif]

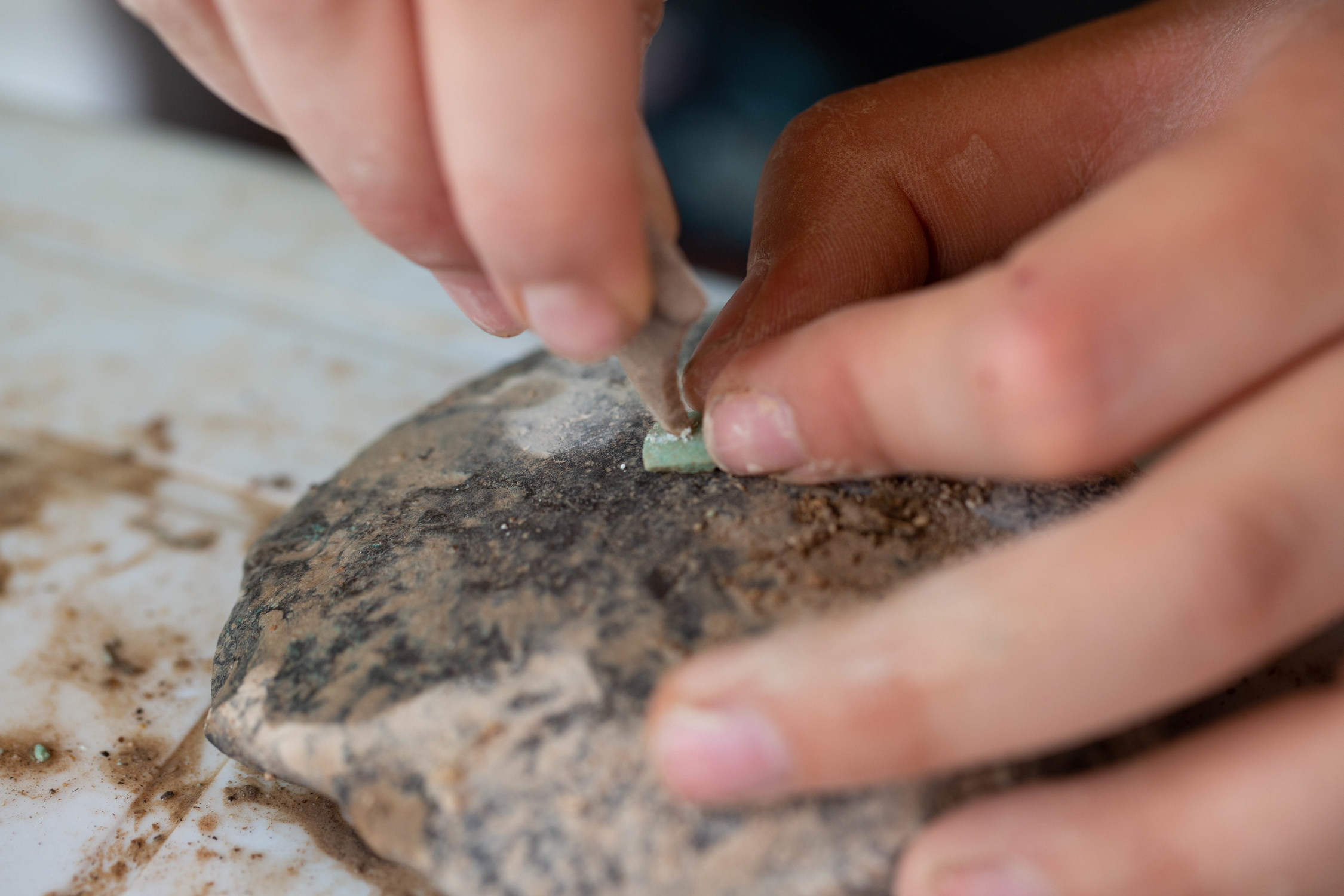

Supplement: S4 Fig — (TIF) [file pone.0292954.s008.tif]

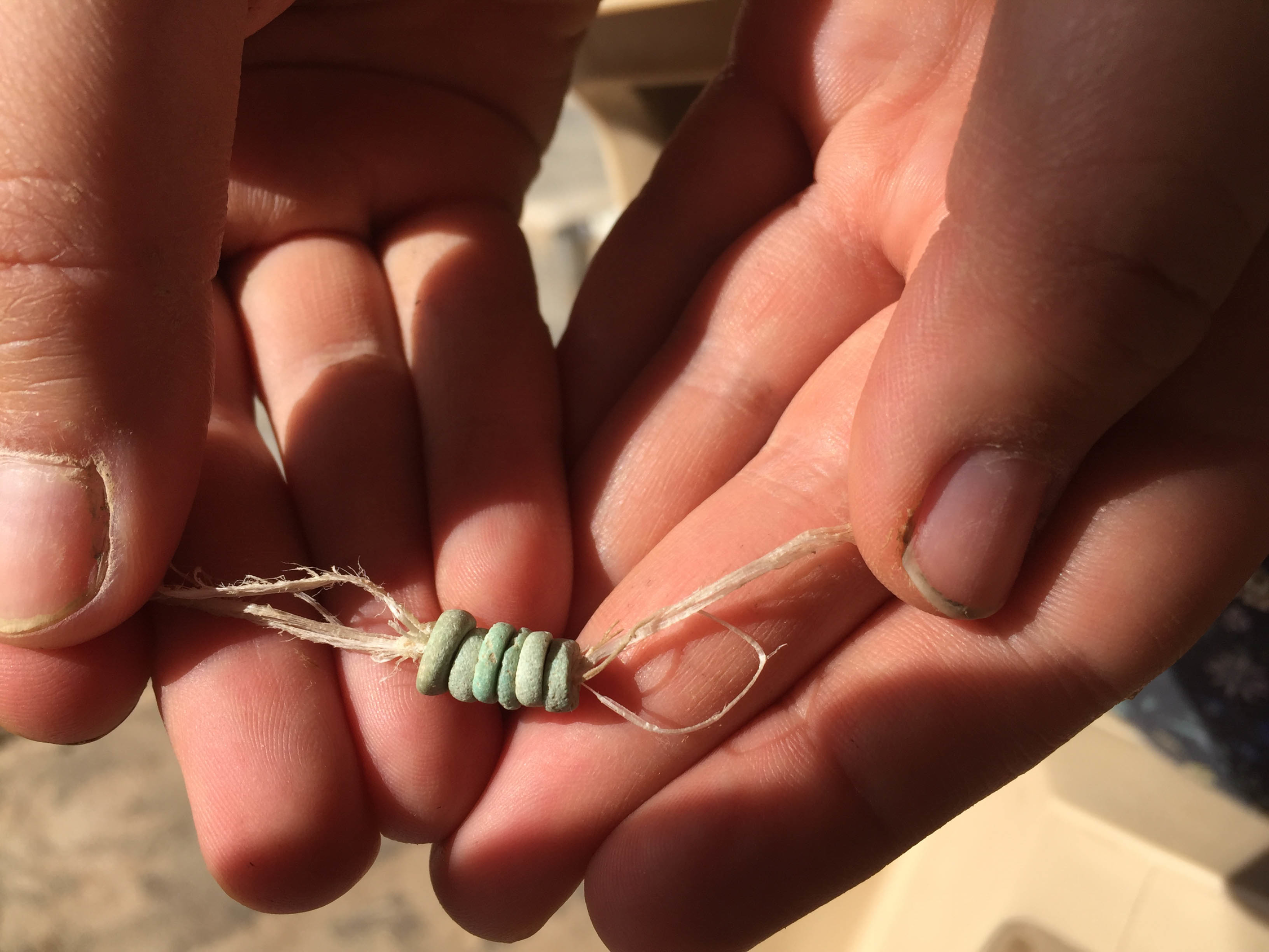

Supplement: S5 Fig — (TIF) [file pone.0292954.s009.tif]

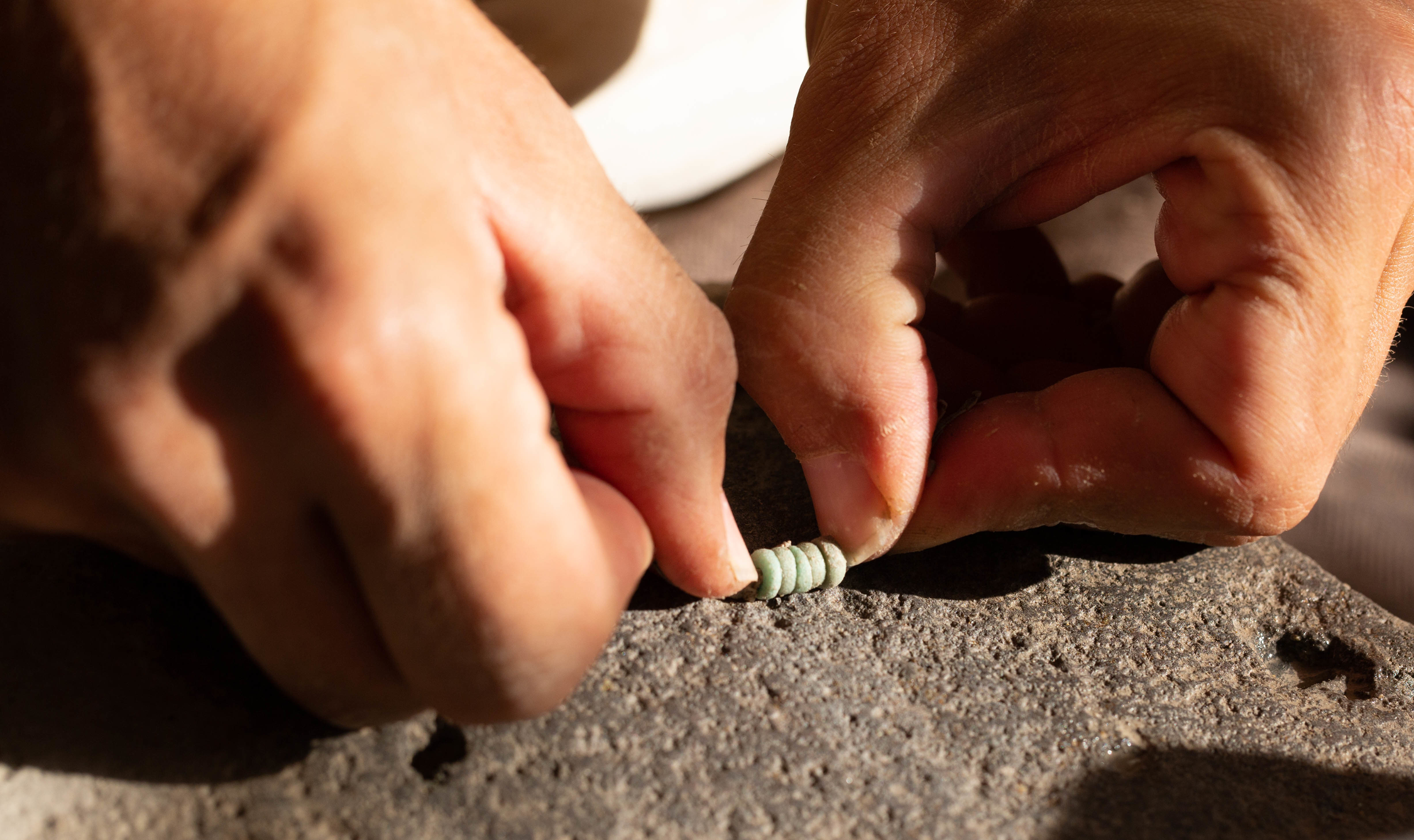

Supplement: S6 Fig — (TIF) [file pone.0292954.s010.tif]

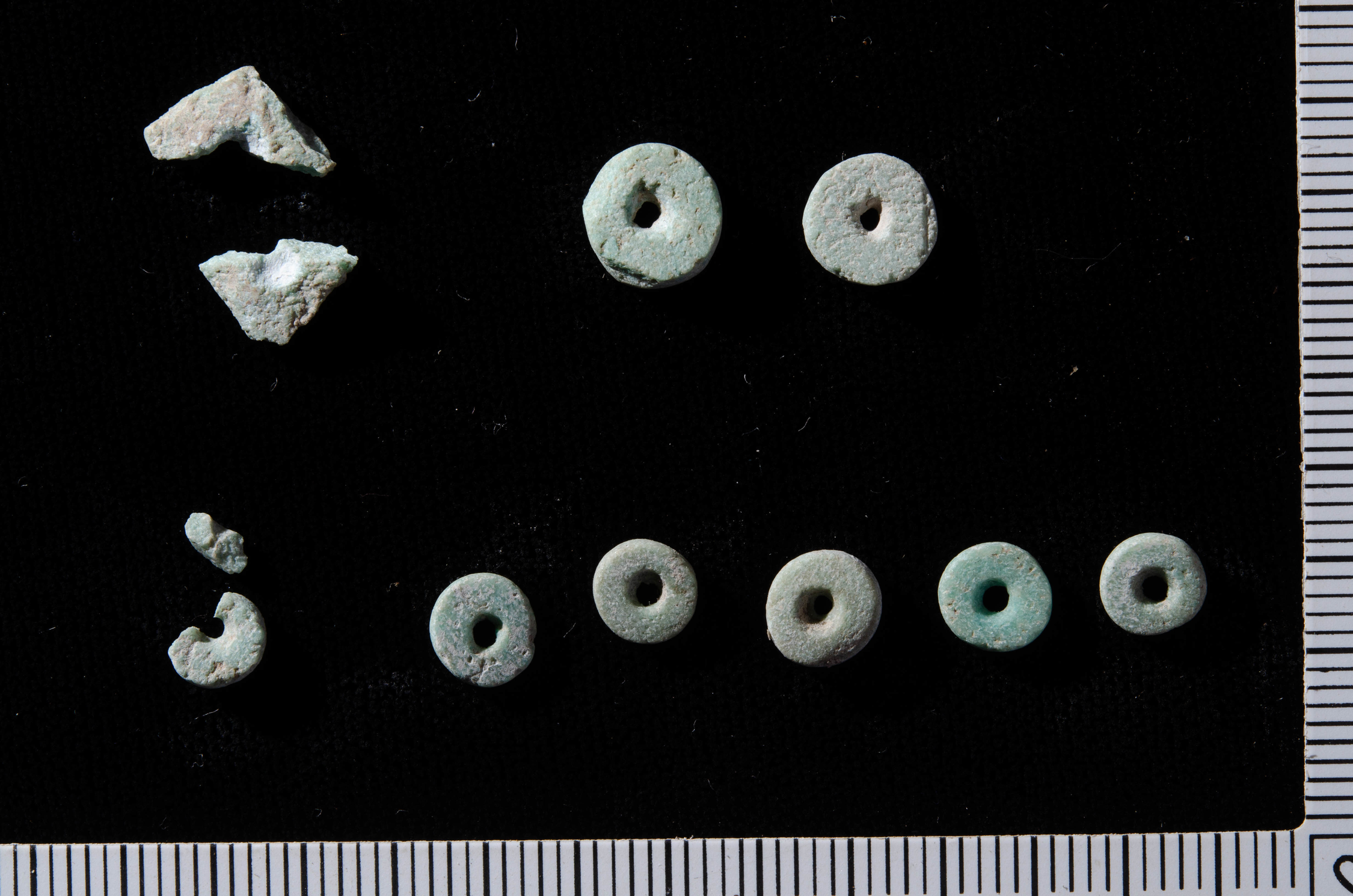

Supplement: S7 Fig — (TIF) [file pone.0292954.s011.tif]
