# Supplementary material for: Nascent craft specialization in the Pre-Pottery Neolithic A? Bead making at Shubayqa 6 (northeast Jordan)
Source: PLoS One. 2023 Dec 8;18(12):e0292954. doi: 10.1371/journal.pone.0292954 (PMC10707568; doi:10.1371/journal.pone.0292954)
Supplement: Attachment — Submitted filename: Response to Reviewers.pdf [file pone.0292954.s012.pdf]

Dear reviewers,

We thank you for your helpful comments, which have aided us in the revision and improvement of this manuscript.

Within the new manuscript you will find that we have made some changes following your suggestions. These include a clarification of the stratigraphic sequence of Shubayqa 6, a more concise description of the geo-chemical analysis, bead typology and production sequence. Some corrections regarding typos and incorrect wording throughout the text have been made, which were rightfully pointed out. We have reorganized the structure of the text in the sense that we have restricted the arguments related to craft specialization to the introduction and to the discussion, so the methods/material and the result section is presenting the evidence as objectively as possible. Although Reviewer 1 is skeptical about the argumentation behind the craft specialization, we still believe that what we consider to be skilled manufacture of disc beads at Shubayqa 6 adds important insights to the wider debate, while clarifying our perception of how craft specialization emerged in early prehistoric societies. Additionally, as we discussed, the significance of household production is traditionally devalued in the discourse aiming at identifying specialization. We do not agree that the evidence is being stretched, as we follow a clear and structured methodology to reach these conclusions.

We have highlighted all the changes within the manuscript, and all comments are addressed in detail below.

On behalf of all the authors,

Mette Bangsborg Thuesen

Review 1

CRITICAL OBSERVATIONS

• There is a certain amount of (unnecessary) repetition within the text. For example, lines 276-279 ("The PPM values ... siliciclastic specimens") repeat text that appears elsewhere; lines 335-343 ("The copper minerals ...light elements") repeats more or less verbatim statements elsewhere in the text (e.g. 322-329). This is unnecessary and should be removed.

Response: This redundancy was due to an editing error. Edited sections were rewritten in the google doc but the original unedited text was not deleted. Therefore, the submitted manuscript contained several uncorrected "ghost paragraphs" similar to their corrected doppelgangers. This has been corrected.

• Insufficient information about the pXRF analyses is provided. Was the instrument used with/without a test stand? In all cases, were measurements made with or without the use of sample pots? What is the degree of statistical precision on the measurements reported in Table S1 – 1 standard deviation, 2SD, 3SD? Note that the abbreviation "Bal" = means "balance" (not ballast) – which is the amount of the signal the instrument is unable to attribute to an element.

Response: We have now updated the text and table. The instrument was used with a test stand but without sample pots. The measurements reported in the table S1 were within a three standard deviation detection limit, standard for the device. The "ballast" typo has been corrected to "balance".

• Moreover, XRF analyzers estimate chemical composition using mathematical procedures (algorithms) to "determine" the relation between the concentration of an element in the sample and the intensity of the fluorescence from that element measured by the instrument. Niton XL3 analyzers offer two mathematical procedures for measurement of geological samples – "Compton correction" (Soils mode) and "Fundamental Parameters" (Mining mode). "TestAll Geo" (note the spelling and capitalization) uses both calibration models and tries to decide which is the best for each element. Unfortunately, the output from the NDT software doesn't report which mathematical procedure (Compton vs FP) was chosen for which element, and in my experience the "Soils" and "Mining" modes can sometimes give quite different results for the same samples. In any case, it is not advisable to trust the compositional data produced by a pXRF analyzer without performing an external calibration check – e.g. by taking measurements on Certified Reference Materials (CRMs) of known chemical composition and comparing the pXRF data with the "known" values (e.g., using linear regression) to derive correction factors (Cal factors) that can be entered into the instrument. Was this done in the case of the Shubayqa 6 samples that were analyzed?

Response: We are confident that TestAll Geo is the most appropriate mode for this analysis. As the reviewer noted, TestAll Geo toggles between the Compton Normalisation and the Fundamental Parameters calibration. However, this is in order to minimise bias arising from differences in the ability of these procedures to measure the total metal content of the sample (Eslami et al., 2020; Lin et al., 2022). TestAll Geo is the most common mode used to analyse

mixed assemblages in archeological contexts, and has been tested against other methods with satisfactory results (Williams et al., 2020).

We have several pXRF analyzers in our lab that are calibrated regularly against CRMs.

Please note that XRF results can often vary considerably from those produced by other methods like ISM-CNR. Were the objective of this study to match precisely archeological specimens to their potential geological quarries we would measure a subset of geological and archaeological samples with ISM and then calibrate the results to our pXRF analyzer. However, the more modest objective of this study as plainly stated, was only to broadly characterise the variability in the assemblage and make a preliminary comparison to a single potential source. Our methods are more than adequate to this end.

References:

Eslami, M., Wicke, D., Rajabi, N., 2020. Geochemical analyses result of prehistoric pottery from the site of Tol-e Kamin (Fars, Iran) by pXRF. STAR: Science & Technology of Archaeological Research 6, 61–71. https://doi.org/10.1080/20548923.2020.1759912

Lin, S.C., White, L.T., Jatmiko, Julianto, I.M.A., Tocheri, M.W., Sutikna, T., 2022. Characterising the stone artefact raw materials at Liang Bua, Indonesia. J Paleo Arch 6, 22. https://doi.org/10.1007/s41982-022-00133-9

Williams, R., Taylor, G., Orr, C., 2020. pXRF method development for elemental analysis of archaeological soil. Archaeometry 62, 1145–1163. https://doi.org/10.1111/arcm.12583

• *In several places in the text (e.g. lines 272-3), it is stated that PPM estimates for 80 elements were produced. This is not possible. At best, Niton XL3 analyzers can quantify up to 43 elements between Mg and U in the periodic table. In practice, the concentrations of some elements are so low that they are below the limits of detection (LOD) of the instrument.*

Response: This was simply an error occurring in several of the “ghost paragraphs” that should have been deleted. The actual number of elements measured was 40. This error has been corrected.

• WAS BEAD PRODUCTION DONE BY SPECIALISTS?
In several places, the authors infer that specialist production is reflected in the high ratio of unfinished goods to finished goods (lines 163, 463-468, 478-481). How so? Bead making whether skilled or unskilled generates a lot of waste – in some circumstances, unskilled production may generate more waste. The ratio of unfinished to finished beads may also be affected by on-site recovery methods – nodules and roughouts are larger than finished beads, therefore more likely to be recovered in sieving. Some finished beads were smaller than the mesh size of the sieves that were used at Shubayqa 6, therefore many beads <5-mm diameter may not have been recovered. If 'dry' sieving was used on site, were sieve residues washed with water before sorting? In my experience, this is essential for recognition/recovery of small artifacts, especially those made of darker colored materials.

Response: “In line 163 we are directly citing one of Costin’s definition of craft specialization. However, in the other sentences, which the reviewer is mentioning here, we are simply stating that the data in combination – and therefore not based on just the ratio of finished to unfinished products – suggest that Shubayqa 6 was a workshop, where bead manufacturing took place regularly given the consistent evidence throughout the different occupational phases. It is therefore this combined evidence that we suggest hints towards what might be considered nascent specialization in bead making.
Although we did have a 0.5 x 0.5 cm mesh size, the soil was often lumpy and would only break up when the material was washed in the field station. We also took bulk sediment samples for archaeobotanic analysis, where the recovery of complete beads was not much higher than those from the dry sieve residue. However, in order to clarify the recovering strategies, we have added more about the sieving strategies during the excavation of Shubayqa 6 to the introduction.”

• *From Figs 3-4 the finished beads don’t strike me as particularly skilled products. I have been involved in several bead-making workshops with University students. After a few hours training/practice, some students can produce results equivalent to what I see in the illustrations here. I don’t accept that “selective use of imported raw material, a standardized production sequence, and an unevenly distributed ratio of roughouts … to finished beads” signifies specialist rather than household production. Stronger evidence is needed!*

Response: "We appreciate Reviewer 1's difference of opinion with regards to the quality of the beads, but disagree with their view. The statement that the beads "don’t strike me as particularly skilled products" appears to us subjective. The argument of the ability of students to reproduce beads is not based on clear experimental protocols and other significant parameters to clarify such as the nature of the raw materials, the technicity of the elements, their sizes, the tools, etc. The comparison between university students making beads as part of their education program or in a controlled experiment setting can in no way be compared to the data we have presented, as no study is cited here that would specify the experimental setup of the bead making workshop referred to here. Some of the students may have already been skilled craftspeople and thus would have had much experience in general craft skills. Further, one cannot compare a set up in which nowadays trained individuals are given a specific goal to accomplish based on accumulated knowledge from archaeological or ethnographic finds , with PPNA hunter-gatherers who also had to find food and cook it, maintain shelters or take care of a myriad of other tasks at the same time. Finally, we do not argue that the Shubayqa 6 bead makers were full-time specialists, as is implied by the reviewers comment that this was household production. What we are proposing here is an alternative production model that does not rely on full-time dedication but is still significant and skilled enough to result in a 'nascent' specialization in stone bead crafts. We therefore stand by our argument.

RECOMMENDATION
That said, the paper has a good deal of merit. It is interesting, well written and presents new data from an important geographical region. But it needs some reorganization and more careful reasoning/argumentation, whist avoiding unnecessary repetition of information and observations. Present the evidence and archaeological background as objectively as possible, reserve consideration of specialist vs household for the Discussion, and draw a reasoned set of Conclusions that do not “stretch” the evidence.

Review 2

The following comments should help the authors to improve their manuscript:
line 62: The period in Late Epipalaeolithic, the culture is Natufian.

Response: “Yes. We have therefore rephrased this sentence slightly, although we would like to avoid using the term culture”

line 65: stone becomes A dominant material, not THE dominant material. Shells are still an important component of ornaments.

Response: “That’s a valid point and we have corrected this line according to Reviewer 2’s suggestion.”

line 114: dates of the Natufian: A date of 15 k now more accepted based on El Wad dating. See https://doi.org/10.1016/j.jhevol.2014.02.011

Response: “We are aware of this article, but as the majority of dates from el-Wad are similar to Shubayqa and cluster around 14,5 kya BP (with only one date falling within the 15 kya BP - and then only at the 2 sigma range), we would like to keep the time frame for the Natufian as already presented in this paper.”

line 118 onwards: This wording of this part is confusing. First give the four major phases, then provide the dates (or present them in a table). Also please present them from older to younger, rather than in stratigraphic order.

Response: “The introduction text has been amended, according to Reviewer 2’s suggestions, The occupational phases are now presented chronologically.”

line 141 – Fig. 2: The caption for this figure should be more detailed, specifying A,B,C,D parts of the figure

Response: “The caption of the figure has been clarified with more detailed descriptions, paying attention to the parts of the beads shown, as suggested by the reviewer.”

lines 144, 148: the numbers of artifacts appear in the results and are not necessary in “materials and methods”.

Response: “The line has been rephrased, following Reviewer 2’s suggestion”

lines 153-4, Fig. 3: what are the five stages? above only four are mentioned (Finished beads, roughouts, debitage and nodules)

Response: “The five stages have been further clarified in the Materials and Method section. They consists of:
1. The extraction of fragments of raw materials

2. The shaping of tabular roughout
3. The drilling of the tabulars
4. The shaping of pseudo-circular shapes of the drilled tabulars
5. The finishing stage of the disc beads."

lines 155-165 do not exactly fit the "methods" section – please consider moving them to the discussion.

Response: "The mentioned lines have been moved and slightly rephrased to fit the start of the discussion, as suggested".

line 178: SI Appendix: please correct :"apetitic limestone" to apatitic (apatite).

Response: "The typo in SI Appendix has been corrected"

line 195: ref 32 should probably be corrected to ref 33. This spelling mistake occurs again in Fig. 7.

Response: "Yes, the reference has now been corrected within the first sentence. However, this reference is not mentioned in Fig. 7, which presents the PPM values from the XRF analysis."

lines 197-8: how are these nine stages (presumably referring ONLY to disc beads) different from the five stages mentioned in fig. 3? Please note that in Wright et al. 2008: Fig. 8, the nine stages contain "perforation error" and "perforation complete" neither of which are a stage on their own. The latter is the result of "perforation 1" and "perforation 2" combined. This is not a stage in itself. Please reconsider. Further down, on line 376 you refer again to five stages, please be more clear about this.

Response: "Yes, we realize that the phrasing may have caused some confusion and have clarified the section that describes the recognized production stages. Early on in this study the production sequence presented by Wright et al. 2008 was used as a reference model, but for the same reasons that the reviewer rightfully points out, we eventually deemed it better to define a new sequence with five stages, which is now the only one described in the manuscript".

line 223, Table 1 and line 234, Fig. 4: 1. It would be useful to include actual numbers in addition to percentages. 2. Because there is no agreed nomenclature of bead types, it would be useful to provide a brief description of the bead types mentioned, or include a plate that shows a drawing from at least two angles (preferably three) with the names. I am not sure I know what "oval" bead or toggle bead are, and what is the difference between tubular and cylinder bead?

Response: "We have added short descriptions for each type into the main text and the actual numbers have been added to Table 2. The figure description for Fig. 4 has been updated as well."

line 302: Calcium Carbonate is CaCO3 (3 in subscript)

Response: “We have corrected this typo.”

line 368: Production sequence: A large portion of this section seems to belong to the discussion rather than results, because it includes many interpretations of the observations.

Response: “We have slightly modified some sentences within the paragraphs. All sentences that are more interpretive in nature, are clearly marked as such (ie. "this suggests” and “this appears”), and we believe that these observations are necessary for understanding the reconstruction process behind the presented production sequence. We still follow clearly the stated methods and therefore their position in the chapter “results” seems justified.”

line 541: "prestige good for elites" is not necessarily evident in the Neolithic. Very likely there were leaders or higher-ranking individuals within the society, but the term elite, at least in archaeology, is more consistent with urban societies. In the next sentences you express a more balanced view of the place of bead making in this society, therefore I suggest to rephrase lines 539-548.

Response: “In this paragraph we refer to former studies on craft specialization that have traditionally focused on urbanized societies. We are therefore not arguing for the presence of elite nor elite goods in the Neolithic.”

lines 564-5: Indeed, seasonality in bead making has already been shown in Palaeolithic sites, e.g., Peschaux, C, G Debout, O Bignon-Lau, and P Bodu. 2017. “Magdalenian ‘Beadwork Time’ in the Paris Basin (France): Correlation between Personal Ornaments and the Function of Archaeological Sites.” In Not Just for Show: The Archaeology of Beads, Beadwork, and Personal Ornaments, edited by D E Bar-Yosef Mayer, C Bonsall, and A M Choyke, 19–38. Not Just for Show: The Archaeology of Beads, Beadwork and Personal Ornaments. Oxford: Oxbow books.

Response: “We thank the reviewer for this reference, which has now been added to the discussion”

lines 567-8: please rephrase last sentence. The link between a and b?

Response: “The sentence has now been rephrased”

line 600: I disagree with the authors that there might be a connection between the Neolithic use of beads as known from the Levant and that of California and other parts of the Americas many millennia later. But the authors' are entitled to their opinion... As with the case of “elites”, I believe that the use of "shell money" is a much later invention that appears after the onset of urbanism, but I do agree that the use of beads in exchange systems was an important aspect of the Neolithic lifeway.

Response: “We understand the reviewer’s point of view, but we would like to keep these references, as we believe that they provide some new ideas into the discussion on the usage of these beads. That being said we do not want to give the impression that we are uncritically making direct analogies to the Shubayqa assemblage, and we have therefore rephrased this

paragraph slightly to underline this."

line 768: Proper citation for Twiss 2015:
In: Susan Pollock (ed.) Between Feasts and Daily Meals. Berlin Studies of the Ancient World 30
www.edition-topoi.de

Response: "This citation has been corrected, thank you."
