# Supplementary material for: Nascent craft specialization in the Pre-Pottery Neolithic A? Bead making at Shubayqa 6 (northeast Jordan)
Source: PLoS One. 2023 Dec 8;18(12):e0292954. doi: 10.1371/journal.pone.0292954 (PMC10707568; doi:10.1371/journal.pone.0292954)
Supplement: Attachment — Submitted filename: Response to reviewers.pdf [file pone.0292954.s014.pdf]

Response to reviewers

We thank the reviewers for taking the time to consider the resubmitted version of our manuscript. Herewith our responses to the comments they have raised.

Reviewer 1)

We have presented data that suggests to us – having examined the entire assemblage directly – that the bead makers at Shubayqa 6 had attained a considerable level of skill in producing different types of beads, stone disc beads being the dominant type. Since there is no universally accepted measure for what being skilled constitutes, we certainly accept that there is always a level of subjectivity engrained in interpretation. Nevertheless, since some of us have analysed several assemblages from the same region and time period, we consider this material to stand out. We suggest a nascent specialization in stone bead craft at Shubayqa 6 based on the following points:

- The assemblage is comprised of a high amount of both finished and unfinished beads in addition to a considerable quantity of raw materials brought to the settlement. We have recently revised the count of approximately 30% of the raw materials used to craft the disc beads at the site. These amounts to a total of 13,695 fragments of varying sizes (we have updated this information in the manuscript). Based on this calculation, we can project that for the remaining 70% of raw materials, the number of fragments could potentially double, if not triple, exceeding 40,000 fragments. This would likely translate to over 7 kilograms of raw materials (while the weight estimation is still in progress, an estimate of 1,700 grams was determined for around 9,000 fragments). Thus far, such a quantity was never recorded elsewhere for this period. Therefore, it is quite reasonable to suggest an important demand for stone disc beads. In this sense, if bead makers of Shubayqa 6 lacked the expertise and the skills to meet this demand, then how should we interpret these numbers?
- We partially concur that from a technological perspective, crafting a disc bead can be within the means of individuals with limited artisanal skills, although this would largely hinge on factors like the nature of the raw material and the quality/adequacy of tools. Nonetheless, our interpretation takes into account the entirety of the assemblage and its archaeological and chrono-cultural contexts. The assemblage has specific characteristics such as a preferred bead type, preferred color, standardisation of the size, and a normalized method of manufacture. It was found alongside a significant quantity of piercing tools and ground stones across all the occupational PPNA levels, thus indicating a constant/continuous production at the settlement. These aspects are persuasive for us to posit that there existed an organizational pattern for crafting stone beads at the settlement. More notably, there seems to have been an intergenerational transmission of know-how. We believe that such a transmission would necessarily involve skilled artisans, probably of distinct degrees of expertise.

Hence, we feel justified in proposing the emergence of specialization in stone bead crafting at Shubayqa 6 involving skilled or highly skilled individuals, while acknowledging that others may

disagree. Following this discussion, we have, however, decided to remove “skilled” from the title of the paper as suggested and also fixed the typographical/grammatical mistakes correctly pointed out by the reviewer.

Concerning the XRF-analysis we feel that the reviewer may have misunderstood the goal and purpose of the analysis, which we carried out. The goal of the pXRF-analysis was to characterize the range of different raw materials and – in those cases where beads were very small – to actually determine whether the material was a form of rock, shell or something else. In other words, we wanted to have some idea of whether the greenstone was:

1) comprised mostly or completely of apatitic limestone (which it clearly was not since copper ores were present) and…

2) to gauge the homogeneity of the greenstone that was comprised of apatitic limestone.

For these objectives the TestAll Geo mode is adequate, and we have consulted on this with other in-house experts in XRF-analysis.

Only three, Wadi Jilat specimens were included in the final comparison (far too few to assess the variance within that quarry) simply to see, where they fell relative to the Shubayqa samples. This was done in order to preliminarily access if this site could have been a source for any of these samples. We did not claim that any of the Shubayqa samples originated there, only that it was an unlikely source for most of them, while it still could not be ruled out as a potential source for some of the Shubayqa specimens. This is a very conservative interpretation, consistent with the methods used.

It is not possible at this moment for us to repeat the XRF component of the study, as the material is no longer available. If the reviewer/editors are unsatisfied with the state of the XRF component of the study, then we suggest taking that component of the paper out of the manuscript altogether.

Reviewer 2)

We thank Reviewer 2 for spotting the misplacement of Timna. We have adjusted the position on the map and are submitting a revised Figure 1.
