# Supplementary material for: Nascent craft specialization in the Pre-Pottery Neolithic A? Bead making at Shubayqa 6 (northeast Jordan)
Source: PLoS One. 2023 Dec 8;18(12):e0292954. doi: 10.1371/journal.pone.0292954 (PMC10707568; doi:10.1371/journal.pone.0292954)
Supplement: Attachment — Submitted filename: TEXT_REVIEWER #1_edited.docx [file pone.0292954.s013.docx]

Revised Manuscript with Track Changes

1	**Nascent craft specialization in the Pre-**

2	**Pottery Neolithic A?**

3	**Bead-making at Shubayqa 6**

**Skilled**

4	**(northeast Jordan)**

5

6

7	Mette Bangsborg Thuesen^1^¶*, Hala Alarashi 2,3&, Anthony Ruter4&, Tobias Richter5&

8

9

10 *corresponding author:
11 E-mail: [mettebt93@zedat.fu-berlin.de](mailto:mettebt93@zedat.fu-berlin.de)

12

13 ¶ These authors contributed equally to this work
14 & These authors also contributed equally to this work

1 Berlin Graduate School of Ancient Studies, Freie Universität Berlin, Germany

2 IMF-CSIC, Barcelona, Spain.

3 Université Côte d’Azur, CNRS, CEPAM, 06300, Nice, France
4 The Globe Institute: Section for GeoGenetics, University of Copenhagen, Denmark
5 Centre for the Study of Early Agricultural Societies, University of Copenhagen, Denmark

# 15 Abstract

16 The emergence of craft specialisation is a key area of interest for archaeologists investigating the socio-
17 economic history and development of past societies. In southwest Asia, as elsewhere, the origins of craft
18 specialisation have been associated with the emergence of surplus food production, households and social
19 stratification. We present evidence for nascent skilled production of green stone beads at the Pre-Pottery
20 Neolithic A (PPNA) site Shubayqa 6, northeast Jordan. Thousands of pieces of debitage, roughouts and
21 finished beads exhibit signs of standardised production that was probably geared towards exchange. This
22 hints at incipient skilled craft production that was likely part-time and seasonal. We therefore argue

towards

23 that the appearance of specialist artisans in this autonomous and non-hierarchical society has no correlation
24 between surplus food production, households, social stratification and craft specialisation.

25

# 26 Keywords

27 Craft specialisation, stone bead production, PPNA, southwest Asia 28

# 29 Introduction

30 The earliest appearance of craft specialists is commonly associated with the emergence of sedentary, surplus
31 food-producing and possibly hierarchical, socially complex societies. Traditional models have emphasised
32 that surplus food production was a necessity for the emergence of ‘serious’ specialists, in the sense of
33 individuals or groups that spend the majority of their time producing standardized objects of high quality for
34 consumption by people other than themselves [1,2]. While this criterion has remained influential in the
35 archaeological literature, today there is a consensus that craft specialisation needs to be understood as a far
36 more complex phenomenon. It is therefore studied as a multivariable continuum that is measurable through
37 group affiliation (independent vs. attached specialists; see Brumfield & Earle [3]; Clark & Parry [4]; Stein
38 [5]), time dedication (part or full-time specialists, see Kerner [6]), the organization of the production
39 (domestic, in workshop, at local or regional levels, see Costin [7] and the quantity or products per specialist
40 and their qualities measured by the degrees of sophistication and standardization [7]. Craft specialisation is
41 furthermore no longer seen as a purely economic activity, but also as a mode of production entangled with
42 social dimensions. In the recent literature, more attention has therefore been given to identifying the social
43 roles of the craft specialists and the use of craft products as active agents in the shaping of symbolic codes
44 shared by different socio-political actors [8–11].
45 As in other parts of the world, archaeologists have generally associated the emergence of craft specialisation
46 in southwest Asia with the rise of socially complex societies. However, several studies have shown
47 that this occurs in various forms throughout the prehistory of this region, stressing that this phenomenon
48 is not restricted to later periods associated with urbanisation. Kerner [6], for example, situated the
49 emergence of craft specialisation in the specialist household production of ceramics during the Early
50 Chalcolithic of the Levant, moving away from a previously dominant focus on metal production [12]. Some
51 have argued that there is already clear evidence for craft specialisation during the PPNB [13], ranging from
52 flint knapping [14] and basketry [15] to plaster production[16]. The appearance of bidirectional naviform
53 blade technology that used non-local, high-quality flint for the production of blades during the Early PPNB
54 has been associated with the potential arrival of traveling craft specialists from the northern Levant [14],
55 where it emerged during the late PPNA[17]. Kirkbride [18] also alluded to the possibility of craft
56 specialisation during the LPPNB, when she described the corridor houses from Level II and III at Beidha
57 as workshops for specialised bone and bead manufacture. Another domain of clear signs of craft
58 specialisation during the PPNB is the production of beads [19–21].
59 The earliest potential evidence for the use of beads in the Levant are naturally perforated *Glycymeris* bivalves
60 from Qafzeh cave dated to around 120,000 cal BP [22,23]. During the Upper- and Epi-Palaeolithic, beads
61 were made from marine and freshwater shells, and animal bones and teeth [24–27]. The Natufian (ca.
62 14,500 – 11,500 cal BP) represents a threshold in the evolution of body ornaments. In addition to little
63 modified shells, bones, and teeth, new types appear with geometric or imitation of natural shapes [28–32].
64 Although the use of stones is attested during this timeframe [33,34], it is during the subsequent PPNA (ca.
65 11,750 – 10,500 cal BP) that stone becomes a dominant raw material for bead production and an important
66 component of the ornamental traditions in Northern Mesopotamia and the Levant [34–36].
67 In the PPNB, another important threshold was crossed, when semi-precious stones were introduced in
68 bead crafting. Stone bead making flourished thanks to unprecedented technological skills that allowed for
69 the transformation of all kinds of stones including very hard, resistant, and delicate varieties into a wide
70 range of bead shapes and types [19,37,38]. This is, for example, evidenced by Groman-Yaroslavski and Bar-
71 Yosef Mayer’s use-wear analysis of two Middle PPNB stone beads from Nahal Hemar cave that revealed
72 the use of lapidary technology, which made them argue that “*The fully developed manufacturing process reflects a*
73 *well-established specialized craft*”[39]. At el-Hemmeh, Raad and Makarewicz [40] also recognised technological
74 strategies in bead manufacturing towards more efficiency in their analysis of the Late PPNA and Late PPNB
75 beads.
76 Wright et al.’s study [11,41] of the Late PPNB/PPNC bead production workshop at Wadi Jilat 13 and 25
77 led them to suggest that the emergence of nuclear households, fully-fledged food-producing economies,
78 and increasing social complexity, resulted in the appearance of specialised bead makers. They argued that
79 beads became an important means to construct and assert social identities towards the end of the PPNB,
80 which fuelled and resulted in the emergence of specialist production centres as well as household
81 specialisation. Baysal [42] argued that although the evidence for specialised production of beads at Bonçuklu
82 Höyük in central Anatolia was not conclusive that we must not assume that craft specialists necessarily
83 resembled fully established artisans during the incipient stages of specialization. While skilled individuals
84 may have spent a great deal of their time producing one type of item intended for exchange or trade, it may
85 be difficult to formally recognize the activities of such individuals archaeologically. More importantly,
86 Baysal [43] highlighted that we should not make *a priori* assumptions under which conditions incipient craft
87 specialists may have become established in the human past, but to focus on the detailed analysis of
88 assemblages and technologies to determine degrees of standardisation, knowledge, surplus production,
89 exchange and the attributed value and meaning of products.
90 In this study, we present evidence for the skilled manufacture of beads at Shubayqa 6 in Jordan, dated to
91 between ca. 12,400 – 10,600 cal BP, thereby making it the earliest lapidary bead production site in the
92 Levant known so far. The occupation spans the transition from the Late/final Epipalaeolithic to the end
93 of the Pre-Pottery Neolithic A (PPNA) and the early Pre-Pottery Neolithic B (EPPNB), a period during
94 which the transition from gathering and hunting to plant cultivation occurred in southwest Asia. During
95 this transition surplus food production was not assured or a given [44]. Although there is evidence for food
96 storage facilities at some sites in southwest Asia during this period [45,46], how significant food surpluses
97 were before the cultivation of domesticated plants is unclear. Despite the recovery of some sickle blades
98 [47] and copious amounts of ground stone tools from Shubayqa 6 [48], no storage facilities or clear
99 archaeobotanical evidence for plant cultivation have been identified to date.
100 Our study provides an unprecedented amount of early evidence for bead manufacturing and suggests that
101 the stone bead production at Shubayqa 6 displays signs of skilled, standardized production that is indicative
102 of nascent craft specialisation. These results highlight how such a production appeared considerably earlier
103 than previously thought, and how it was not necessarily connected to surplus food production. We
104 therefore argue that alternative scenarios for the emergence of craft specialisation in southwest Asia should
105 be considered.

106

## 107 Shubayqa

108 Shubayqa 6 is located in the volcanic lava field of the *Harrat al-Sham* (Black Desert) in Northeast Jordan,
109 ca. 130 km east of Amman (see Figs 1 and 2). The settlement is situated at the northern edge of the Qa’
110 Shubayqa mudflat from which the site takes its name. The area has produced evidence for a dense
111 concentration of late Pleistocene and early Holocene sites dating from the Early Natufian (c. 14,500 –
112 14,000 cal BP) to the end of the PPNA (c. 11,600 – 10,500 cal BP.). The site of Shubayqa 6 forms a
113 settlement mound measuring ca. 3.000 m^2^ in an area that rises ca. 1.5 – 2 m above the surrounding area.
114 Excavations have to date focused on a 10 x 10 m large area on the east side of the mound. Radiocarbon
115 dates, chipped stone artefacts, and stratigraphy currently suggest occupation stretching from the end of the
116 Epipalaeolithic to the end of the PPNA and the EPPNB. The site has three major occupation phases,
117 distinguished on the basis of the site’s stratigraphy, lithic typology and radiocarbon dates. The earliest
118 occupation is a final Epipalaeolithic/Khiamian phase, characterised by the presence of both short lunates,
119 el-Khiam points and many drills. The middle phase dates to the early PPNA and is characterised by fewer
120 lunates (compared to the preceding phase), some el-Khiam points, and many drills. The last and uppermost
121 phase dates to the late PPNA/early PPNB, with a chipped stone assemblage characterised by a greater
122 variety of points - including Helwan points - with lunates being virtually absent. The earliest phase, final
123 Epipalaeolithic/Khiamina, was radiocarbon dated to 12,300 - 11,800 cal BP (all dates reported here are the
124 summed calibrated dates for each phase at 68.2% confidence, calibrated in OxCal 4.4. [49] using IntCal20
125 [50]. The early PPNA phase is dated to ca. 11,900 - 11,200 cal BP, while the late PPNA/early PPNB phase
126 dates to 11,090 - 10,600 cal BP. The Khiamian and EPPNA phase was uncovered in the main excavation
127 area and is associated with two larger oval, stone-built structures (Space 1 and Space 4) with stone paved
128 floors, as well as some smaller adjacent structures. The LPPNA/EPPNB phase is associated with
129 two fully excavated smaller, oval buildings with trampled floors (Space 3 and Space 10), and several other
130 partially excavated buildings. Finds include chipped stone tools and waste from chipped stone manufacture,
131 numerous ground stone artefacts, worked bone and faunal remains, as well as botanical remains. Current
132 evidence suggests that the site was intensively occupied and that people spent extended periods at
133 the site. Although one should be cautious with the application of specific terms such as ‘village’ [11,50:
134 p.79-81], Shubayqa 6 should probably not be understood as a transitory camp, but as a place in which a community
135 invested considerable time and resources. Excavations were carried out using the single-context
136 recording method. Deposits and layers spread over a larger area, e.g. middens, deposits above floors, were
137 excavated in a 1 x 1 m grid system using arbitrary excavation spits. All excavated sediments were dry-sieved
138 on site using 0.5 x 0.5 cm mesh. The dry-sieve residue was collected, washed, and sorted in the field
139 camp. In addition, bulk sediment samples were collected for archaeobotanical flotation, and all ‘heavy
140 residue’ collected in the flotation tank was washed and sorted in the field station. Thus, we are certain of a
141 high recovery rate of even small beads and bead fragments.
142 **Fig 1. Map with the location of Shubayqa 6 and mentioned sites.** Map created using data from
143 ©OpenStreetMap using ©OpenTopoMap style under license CC-BY-SA in QGIS Vs. 3.28.
144 **Fig 2. Plan of the excavation at Shubayqa 6.**

145

# 146 Materials and method

147 The studied assemblage comprises nearly 2500 beads and bead roughouts from Shubayqa 6. In addition to
148 finished beads and roughouts, the dry sieving of all excavated sediment, as well as extensive sampling of
149 sediment for flotation, resulted in the recovery of large amounts of greenstone debitage and raw material
150 nodules. This material has thus far not been fully quantified, but initial analysis of a selected group revealed
151 more than 9000 fragments of production waste with a substantial amount still remaining to be counted.
152 Most of the material is from midden deposits that accumulated inside Space 1 and 4. Finished beads,
153 roughouts, debitage, and nodules were recovered from all excavated contexts, although there are clear
154 concentrations associated with Spaces 1 and 4 (EPPNA), and Space 3 (LPPNA). Spaces 2, 5, 6, and 7
155 contained relatively few beads and these mostly included finished disc beads and disc bead roughouts.
156 In this study, the analytical method is based on the *châine opératoire* approach [52,53] implemented on
157 beads (e.g. Alarashi [54]; Barthélémy de Saizieu and Bouquillon [55], where we examine the diversity of
158 raw material used and their potential sources, the production sequences, and – to a lesser extent – a
159 general assessment of use-wear of the finished stone beads. The analysis of the production sequence
160 relied on recording of quantitative and qualitative data. The latter was based on a classificatory approach
161 that categorised artefacts into different production stages. Unfinished beads were registered as roughouts
162 while finished beads were further sub-divided into distinct typological categories following the
163 classification system developed by Wright & Garrard [11] supplemented in some cases by additional
164 categories drawn from Beck [56], Bains [57], and Bar-Yosef Mayer [33]. The vast majority is defined as
165 disc beads in both finished and unfinished forms.
166 Among the typological diversity, only disc beads occur in different stages before the final products. This
167 allowed the reconstruction of their production sequences and the identification of their order (Fig 3).
168 Partially inspired from previous works [41], a total of five manufacturing stages were established:
169 1. The extraction of fragments of raw materials (nodules);
170 2. The shaping of tabular roughout (tabulars);
171 3. The drilling of the tabulars (drilled tabulars);
172 4. The shaping of pseudo-circular shapes of the drilled tabulars (unfinished disc beads);
173 5. The finishing stage of the disc beads (final products).
174 **Fig 3. Greenstone roughouts and beads recovered from Shubayqa 6 divided according to the five**
175 **identified production stages.**
176 The macroscopic observations were supplemented by microscopic analysis to record more minute
177 production traces, such as drilling and abrasion patterns. The variables for the microscopic analysis were
178 defined according to the same system used by Bains [57] for her studies on the stone beads from
179 Çatalhöyük that include drilling characteristics, perforation morphology, characterisation of perforation
180 marks, as well as marks on the length/height (the profile of the bead) or the ends (perforated surfaces) of
181 the beads, shape of edges, and evidence of use. A total of 110 stone beads were sampled for this part of
182 the analysis, conducted using a Brunel SP-400 incident light microscope and a GXM MZSTLED
183 stereo microscope. The specimens were selected based on stratigraphic context to ensure that a
184 representative sample for each occupation phase and production stage was studied.
185 These observations were supplemented by a small experimental program for which the production of
186 greenstone beads was replicated during the 2019 Shubayqa field season to further test different methods
187 and techniques (S2 Appendix).
188 Because the procurement of raw materials is a significant component of the labor invested in production,
189 we sought to describe the variance in raw materials used for beads to infer something about their raw
190 material and potential sources. To this end, we selected 33 beads from the Shubayqa 6 assemblage for an
191 amended (non-destructive) petrographic analysis, utilizing stereomicroscopy to describe mineralogy and
192 texture and a NITON XL3 GOLDD+ pXRF energy dispersive x-ray analyser to estimate elemental
193 composition. Three additional greenstone samples from the Wadi Jilat, a known prehistoric quarry were
194 also analysed to make a preliminary comparison with the apatitic limestone specimens from Shubayqa.
195 The composition in parts per million (PPM) of 40 elements plus balance (Bal), the amount of the signal
196 that the instrument is unable to attribute to an element (roughly proportional to the sample mass of the
197 unmeasured light elements), was generated for each of the 36 samples plus two modern ostrich eggshell
198 controls. All measurements were made at standard room temperature and pressure (approx. 1 bar)
199 without helium purge or vacuum, using a test stand. The specimens were cleaned with deionized water
200 and measured directly without sample pots. The resulting data were processed with “Standard Thermo
201 Scientific™ Niton Data Transfer (NDT™) PC software suite” set for “TestAll Geo” at four separate
202 runs of: light elements, medium heavy elements, heavy elements, and standard elements each measured for
203 120 seconds.
204 The principal objective of this part of the study was to assess the diversity of the assemblage by gauging
205 how many different types of materials were used in the bead production at Shubayqa 6 and to measure
206 the degree of homogeneity in the ‘greenstone’ category. This limited exploratory characterization is also
207 an initial step towards the identification of raw-material sources, but that is beyond the scope of this
208 study.

209

# 210 Results

211 We recorded 2398 beads and bead roughouts from Shubayqa 6 as part of this study. Of the finished and
212 unfinished stone beads, which we focus on here, 533 were recovered from early PPNA (EPPNA) contexts,
213 501 from late PPNA (LPPNA) contexts. 1307 came from topsoil or disturbed contexts and are thus less
214 secure. Since this bead-making technology is quite specific to the Pre-Pottery Neolithic, and since there was
215 no occupation at the site between the start of the EPPNB and the Early Bronze Age we feel confident that
216 this material is broadly related to the PPNA occupation (see **Table 1**). Roughouts make up the largest single
217 group accounting for 50.4% in the EPPNA and 53.7% in the LPPNA phases. The most common type are
218 disc beads, which account for 36.6% and 42.1% of the beads in the EPPNA and LPPNA phases
219 respectively.
220 **Table 1. Heat map correlating the bead typology with percentage distribution across the**
221 **occupational phases. Actual numbers are marked in brackets.**

| **Bead type** | **EPPNA** | **LPPNA** | **Post-Neolithic/Mixed** |
| --- | --- | --- | --- |
| Disc beads | 42,1% (197) | 36,61% (157) | 32,26% (342) |

| Oval beads | 0% | 0% | 0,08% (1) |
| --- | --- | --- | --- |
| Tubular beads | 3,81% (20) | 6,75%% (33) | 2,13% (27) |
| Cylinder beads | 0,38% (2) | 0,2% (1) | 0,16% (2) |
| Barrel beads | 1,52% (8) | 0,2% (1) | 0,71% (9) |
| Pendants | 0,19% (1) | 0,82% (4) | 0,16% (2) |
| Double perforated  beads | 0,19% (1) | 0,2% (1) | 0,47% (6) |
| Toggle beads | 0% | 0% | 0,08% (1) |
| Perforated pebbles | 0% | 0% | 0,16% (2) |
| Indeterminate | 0,57% (3) | 0,2% (1) | 0,55% (7) |
| Roughouts^a^ | 51,24% (269) | 55,01% (269) | 63,25% (802) |

222 aroughouts represent preforms of disc beads.

223

## 224 The finished beads

225 Among the studied assemblage 931 beads were assigned to a specific type, while the remainder were
226 classified as roughouts (n=1340). The most common types are shown in Fig 4. The majority of beads
227 (n=809) belong to the disc beads that are characterised as flat beads of circular shape. Although these were
228 predominantly made from stone, other kinds of raw material, such as ostrich eggshell, bone and clay, were
229 also used. Other common bead stone types include barrel beads, cylinder beads (cylindrically shaped with
230 narrow perforation), double perforated beads (flat beads of rectangular shape), tubular beads (cylindrically
231 shaped with wide perforation), and pendants (triangular or trapezoidal shaped beads) although they are
232 comparatively rare and all made from greenstone. Finally, oval beads (flat beads of oval shape), toggle beads
233 (dumbbell shaped) and perforated pebble were each represented with just 1-2 specimens.
234 **Fig 4. Most common types of stone beads found among the Shubayqa 6 assemblage.** (A) Side view
235 of disc beads of various sizes with broken roughout (B) barrel bead with profile and both ends shown (C)
236 profile of tubular bead (D) one end of tubular bead.
237 To assess the degree of standardization in the Shubayqa 6 disc bead production, we measured overall bead
238 diameter and maximum thickness of all complete beads from secure EPPNA and LPPNA contexts (N=
239 96). The result (**Fig 5**) indicates that there is no significant size difference for EPPNA or LPPNA disc
240 beads. The smallest bead measures 3 mm in diameter with a minimum thickness of 0.8 mm. There is a clear
241 clustering between 3 – 6 mm diameter and 0.5 – 1.5 mm in thickness. Beads with greater diameter and of
242 greater thickness exist from both phases, but more so from the EPPNA, while LPPNA beads cluster more
243 tightly. However, this does not necessarily suggest greater standardization, as overall sample sizes are too
244 low to make this inference. Nevertheless, the clustering of EPPNA and LPPNA beads does suggest that
245 bead makers worked towards a specific size template of small and thin beads. In this sense, the Shubayqa
246 6 bead production can be seen as standardised during both the EPPNA and LPPNA.
247 **Fig 5. Scatter plot of the PPNA disc beads from Shubayqa 6.** The general size is represented by the
248 relation of the diameter and the thickness. Lineal regressions of the EPPNA and LPPNA beads show
249 similar distributions with a slight smaller size of the LPPNA.
250 The majority of beads examined under the microscope did not show traces of use. Only five finished beads
251 displayed wear marks, including widened holes in the perforated areas and unevenly smoothened surfaces
252 on the ends of the beads, which could derive from the beads being worn. Therefore, there is at present little
253 evidence of use-wear, but given that the sample size was small, future work may provide more results.

254

## 255 Raw materials

256 Of the 2398 beads recovered, 1897 (79.1%) were made from rocks and minerals, while the remaining 20.9%
257 were produced from hard animal tissues, namely ostrich eggshell, marine shells and animal bones (see Fig
258 6). Among the first group, 72.8% were classified as “greenstone”, a category that in the Levant includes
259 different types of greenish rock and minerals such as apatitic limestone varieties, malachite, turquoise,
260 amazonite and copper silicates [58]. Other 6% of the total number of the beads from this group could not
261 be classified yet, while less than 0.5% were identified as basalt, flint, clay, and ochre.
262 **Fig 6. Distribution of raw material according to the bead typology.**
263 This basic classification is augmented by the XRF analysis of selected specimens from Shubayqa and
264 geological samples from the Wadi Jilat. Fig 7 lists PPM values from each sample for eight of the 40 elements
265 measured in the XRF analysis. These elements were selected for the table because they are major
266 constituents and differ significantly between the material classes. The PPM values of balance (Bal) are also
267 listed. High concentration of both calcium (Ca) and phosphorus (P) in most of the greenstone specimens
268 as well as the samples from Wadi Jilat, confirm that these are apatitic limestone, consistent with the green
269 Dabba Marble identified in other Neolithic contexts in the southern Levant [11,41]. In contrast, calcite and
270 eggshell specimens are distinguished by high concentrations of calcium alone. High concentrations of
271 copper (Cu), as well as manganese (Mn), distinguish the copper minerals, while the mass of the siliciclastic
272 specimens are largely comprised of silica (Si) and other clay minerals including aluminium (Al) and
273 potassium (K). The PPM estimates for 40 elements plus balance generated for each sample were used to
274 generate the Principle Component analyses presented in Fig 8 (A and B).
275 **Fig 7. lists PPM values from each sample for eight of the 40 elements measured in the XRF**
276 **analysis.**
277 Fig 8A plots the Principal component analysis (PCA) scores of all 36 samples on the first and second
278 components calculated on the variance-covariance matrix in which the influence of each element is
279 equivalent to its compositional proportion. This is useful for sorting very different classes of material (e.g.
280 limestone and metal ores and siliceous rock).
281 **Fig 8A. The PCA biplot generated from the XRF analysis of all Shubayqa specimens and several**
282 **from Wadi Jilat along with two modern ostrich eggshell controls.**
283 The apatitic limestone specimens generally plot from the center to the upper right quadrant due to their
284 concentrations of both calcium and phosphorus. A second adjacent cluster in the lower right is formed
285 from samples that share the high concentration of calcium but have relatively less phosphor, consistent
286 with their identification as precipitated carbonate rock (probably calcite) and ostrich eggshell. A third cluster
287 is distinguished by relatively high concentrations of silicon (Si), aluminium (Al), potassium (K) and iron
288 (Fe) and very little calcium (Ca), consistent with their petrographic designations of siliciclastic rock. These
289 including sandstones, silicified mudstone or slate, and hard crypto-crystalline silicate rock, which could
290 occur as a precipitate within a limestone facies.
291 Two of the 36 specimens (Shub-2a and Shub-23) analysed with XRF were distinguished by relatively high
292 copper content (587,095 and 327,396 ppm Cu respectively), which is well within the range of commercial
293 copper ores. This coupled with the relatively low concentrations of sulphur (S), iron (Fe), and phosphor (P),
294 make malachite, which is a copper carbonate hydroxide mineral, the most likely candidate for both
295 specimens. However, chrysocolla cannot be ruled out for the second sample (Shub-23) because both silicon
296 (Si) and aluminum (Al) are significant constituents. Specimen Shub-2a has a significantly higher copper
297 content (587,095 ppm), yet no trace of barium or lead consistent with relatively pure malachite, which does
298 outcrop in the Wadi Faynan and Wadi Fidan area. The significant variance in these trace elements might be
299 used to match the two copper-bearing specimens to their respective ore sources, when a reference database
300 is available.
301 Fig 8B compares the composition of Shubayqa specimens designated as greenstones along with three
302 apatitic limestone samples from the Wadi Jilat by plotting the sample scores of 40 elements measured with
303 XRF of the first and second components, which explain 32.8% and 21.3% of the total variance respectively.
304 The PCA is calculated from the correlation matrix, which assigns equal weight to all elements, including
305 those with “trace” concentrations. This is useful when comparing archaeological material to geological
306 samples from potential sources. As noted above, the copper minerals are again clearly distinguished by their
307 relatively high concentration of copper as well as potassium (K), aluminium (Al), titanium (T), lead (Pb),
308 iron (Fe), manganese (Mn) and magnesium (Mg) relative to calcium (Ca) and phosphorus (P) comprising
309 the limestone specimens. Variability in the Shubayqa limestone is driven by a suite of elements including
310 antimony (Sb), tin (Sn), chromium (Cr) and strontium (Sr). Samples from Wadi Jilat are homogeneous and
311 cluster close to a subset of Shubayqa samples relatively enriched in these elements along with unmeasured
312 light elements. This cluster has less of the trace elements tungsten (W) and molybdenum (Mo) as well as
313 the principle constituents of apatitic limestone, calcium and phosphorus. Although we have far too few
314 specimens to adequately describe the variability of the Wadi Jilat source, this preliminary analysis suggests
315 that while some of the Shubayqa limestone may have originated there that was not the case for the majority
316 of samples. The limestone at Shubayqa is variable and was probably sourced from several different quarries.
317 **Fig 8B. The PCA biplot calculated on the correlation matrix generated from the XRF analysis of**
318 **the Shubayqa specimens designated as greenstones along with three apatitic limestone samples**
319 **from the Wadi Jilat.**
320 In summary, The XRF analysis showed that most of the greenstone at Shubayqa 6 was Dabba Marble,
321 although other types of greenstone are also present in the assemblage. The analysis also identified copper
322 ores, which do not occur in the Azraq Basin or Harra. Other minerals used for bead making appear to be

323

travertine, sandstone, and silicified mudstones. Most of the stone samples that were studied

XRF did

324 not measure more than 3.5 – 4 on Mohs hardness scale and are therefore considered soft stones that are

for

325 easy to work. Thus, artisans clearly preferred soft stones with greenish or reddish colour.
326 Some of the (Dabba Marble) may have been mined near the Wadi Jilat in the PPNB [11,41] located ca. 130
327 km distance from the Qa’ Shubayqa. Other outcrops of greenstone are known from the Azraq oasis situated
328 ca. 75 km away, and Dabba Marble sources have also been found in the Wadi al-Qattafi area in the
329 southwestern part of the *Harra* (Gary Rollefson, personal communication). The presence of raw material,
330 debris and debitage shows that larger fragments were brought to the settlement and manufactured there.
331 These unworked greenstone pieces usually measure between 1-8 cm and were therefore easily portable.
332 Analysis conducted as part of this study was too limited in scope and therefore cannot pinpoint the exact
333 place of origin, but since there are no known sources of “greenstone” in the volcanic lava fields of the
334 *Harra*, it is clear that the raw material was imported to the site over medium to long distances. We therefore
335 consider the central, western and southwestern Azraq Basin as the most likely sources of most of the stone
336 raw material found at Shubayqa 6. Sources for some of the rarer materials, such as malachite and other
337 types of greenstone, can be found in the Wadi Faynan/Fidan area in southern Jordan, located ca. 250 km
338 away [59,60]. Although the scope of the analysis carried out in the present study is limited and should be
339 expanded, these initial results suggest that the inhabitants likely obtained some raw material from nearer
340 sources, while receiving either raw material or finished beads from further afield.

341

## 342 Production sequence

343 Using the sequential classification system of Wright et al. [41], it was possible to discriminate the frequency
344 of different production stages among the disc beads in the Shubayqa 6 assemblages. A production sequence
345 was established that consists of five main stages, which are illustrated in Fig 3. At stage 1 the roughout is
346 an unworked primary blank, which at the next step, stage 2, is initially shaped by abrading the ends to make
347 a more even surface. At stage 3 the roughout is perforated, which was done by drilling from both sides.
348 Some of the studied roughouts assigned to this stage showed perforation errors caused by a misalignment
349 of the biconical perforation. The next stage (4) represents the nearly completed bead, which besides full
350 perforation had also been polished into a near-final bead shape. Stage 5 marks the concluding stage of
351 production, when the surfaces of the bead had been completely smoothened.
352 The greatest range of production stages was recorded for beads made from greenstone, as every step is
353 represented among this group. Disc beads made from other kinds of stone are only represented by later
354 production stages (3-5), suggesting that they were either not made on site or only finished off there. We
355 therefore focus on the greenstone (Fig 9). Greenstone roughouts from stage 1 are very rare (0.1%).
356 However, an initial study of the greenstone debitage and debris suggests that many of these had been initially
357 sorted into the unworked fragments. It is therefore expected that further analysis of the debitage will
358 increase the number of stage 1 roughouts. The fact that greenstone disc beads are well-represented in every
359 production stage together with the evidence of raw material import, suggests that their entire production
360 cycle took place on site. There is little apparent change in this pattern between the EPPNA and LPPNA
361 phases, and the data from mixed or post-Neolithic contexts also shows a similar pattern. There is also some
362 evidence for the re-use of broken pieces, such as roughouts with misaligned perforations that were flattened
363 or reshaped, so they could still be threaded (See Fig 10).
364 **Fig 9. Percentage distribution of production stages according to the occupational phases at**
365 **Shubayqa 6.**
366 **Fig 10. Roughout that was flattened after breakage in order to still allow threading.**
367 The microscopic examination disclosed more information about the production techniques. About 15 %
368 of the sampled stone beads showed abrasion marks on the profile and the surfaces of the perforation,
369 suggesting that grinding was the main method to shape roughouts. This was done in one recurrent motion
370 in the same direction. When it comes to the perforation stage the majority of the sampled beads appeared
371 to have been drilled from both sides (biconically). The assessment of the perforation morphology
372 furthermore showed that the angle of the perforation was often slanted/uneven rather than straight (see
373 **Fig 11**). This resulted in occasional perforation errors, as evidenced by the finds of roughouts where the
374 drilled holes were completely misaligned.
375 **Fig 11. Results of the microscopic analysis observing drilling technique, based on the shape of the**
376 **perforation.**
377 Wright et al. [41] observed two drilling methods in the Jilat bead assemblage: hand drilling using a borer or
378 piercer, which was either held in the hand or hafted on a drilling stick, and mechanical drilling using a bow
379 drill secured with a capstone. Both methods result in different perforation shapes and marks. A concentric
380 shape and parallel-circular wear marks resulted from mechanical drilling, while slanted perforation shape
381 and non-parallel, coarse perforation marks are related to drilling by hand (see also Bains 2012 [57]). The
382 analysis of the perforation marks in the Shubayqa 6 assemblage was not conclusive, as most of the analysed
383 beads had rough or indeterminate marks. Faint concentric striations, which indicate drilling by mechanical
384 means, were primarily present in unfinished pieces on the interior sides of the perforation. Meanwhile
385 smooth perforation marks appeared on seven out of nine finished pieces. However, these can also be caused
386 by the final stage of polishing if the beads were strung together. In the Shubayqa 6 assemblage, three pieces
387 exhibited traces indicating mechanical drilling, while 15 pieces had traces indicative of hand drilling. In the
388 case of the other beads we examined, the outline of the perforation was often regular, but accompanied by
389 slanted perforation morphology and coarse perforation marks. We interpret this combination of wear traces
390 as drilling by hand.
391 When it comes to the final shaping, most of the finished beads had smoothed surfaces, in contrast to
392 roughouts which had rougher abrasion marks, suggesting that the beads were polished during the last stage
393 of production. The morphology of the edges, which were only observed on finished beads, also showed a
394 clear correlation between the type of bead and the shape of the edge. The disc beads predominantly featured
395 sharp edges, while the more uncommon types, such as barrel beads and double perforated beads all have
396 rounded or a mixture of sharp and rounded edges. This suggests that disc beads were polished together *en*
397 *masse* and other types were smoothened individually [57].
398 To summarise, the *chaîne opératoire* for disc greenstone beads at Shubayqa 6 can be reconstructed as follows:
399 greenstone raw material was obtained either directly or through exchange in areas away from the *Harra*.
400 Nodules were brought to the settlement, where they were split by percussion. These blanks were then
401 further reduced to roughouts by chipping and flaking. Wright et al. [41] suggest that this would have been
402 possible using indirect percussion, soft-hammer percussion and pressure flaking. No retouch marks
403 from this process were recognised on the material in the Shubayqa 6 assemblage so the exact technique is not
404 distinguishable based on the currently available data. Nevertheless, our experiments (S2 Appendix) indicate
405 that the best way of creating flakes was by cutting grooves in the greenstone nodule and then knapping it,
406 while it was being wedged. In some cases, saw marks were recognised on a few specimens suggesting that
407 occasionally blanks were made by sawing regular lines halfway through the flake and then it could be almost
408 effortlessly snapped into pieces. The experimental work further showed that this was a highly efficient way
409 to produce square shapes, and the microscopic studies confirmed that this was the most commonly used
410 technique by the Shubayqa 6 bead makers. Abrasion marks indicate that bead roughouts were often ground
411 on an abrasive surface until they took on a square shape. This was likely done using a grinding tool of basalt,
412 of which there are many at the site [48].
413 Once the roughout had been shaped into an approximate square the drilling of the central perforation
414 commenced. This choice appears to have been made to streamline the production process: since the drilling of
415 the fragile disc bead roughouts carries a high risk of accidental breakage, it was done early in the process to
416 avoid having to spend too much time on a single bead that might subsequently break during drilling. In
417 addition, early drillings allow stringing several roughouts together in order to apply batch or *en masse*
418 polishing. This appears to be an efficient way to reduce the overall time needed for production and
419 highlights the experience and know-how of the Shubayqa bead makers. This process was reversed for
420 pendants, as preforms of these indicate that drilling was the last part of the process for these types, but as
421 these were also manufactured in larger sizes the risk of breakage during drilling was lower than
422 for the small disc roughouts.
423 The majority of the sampled beads had biconical perforations, meaning that the beads were drilled from
424 both sides using a borer or piercer, which was either held in the hand or hafted on a drilling stick. Our
425 experiments showed that biconical drilling was the most successful strategy to avoid breakage: uniconical
426 perforation often resulted in too much pressure which led to breakage. This work furthermore revealed
427 that it was advantageous to have rectangular roughouts, because they made it easier to centre the perforation
428 ensuring that holes were aligned (see figures in S2 Appendix). Preliminary analysis of the chipped stone
429 artefacts from Shubayqa 6 shows that drilling tools make up ca. 20% of the tool assemblage from EPPNA
430 and LPPNA contexts (see Fig 12). Although functional analysis on these tools was not performed yet, their
431 high frequency is likely related to bead production at the site. Our own experiments provided no clear
432 evidence that production time was significantly reduced when abrasives (sand and water) were added during
433 the drilling. Once perforated the roughouts were abraded one final time. Based on the uniformity in the
434 size of the beads and the sharp edges, the disc beads were probably strung together and abraded in a cyclical
435 motion until they had smooth surfaces and reached their final shape [57]. This stage was also successfully
436 replicated during the experimental part of the analysis that produced beads of the same size with sharp and
437 polished edges. When we calculate the ratio of finished to unfinished beads (roughouts), unfinished beads
438 always outnumber finished ones slightly (see Table 2). The large amount of debitage from bead manufacture
439 in all occupation phases at Shubayqa 6, as well as the high frequency of tools involved in bead making
440 within the same areas underlines that bead-making took place regularly.
441 **Table 2. Ratio of finished beads in comparison to roughouts.**

|  | Total amount of beads | Complete beads/versus roughouts |
| --- | --- | --- |
| EPPNA | 533 | 1 : 1.02 |
| LPPNA | 501 | 1 : 1.16 |
| Mixed/Post-Neolithic | 1307 | 1 : 1.58 |

| Average |  | 1: 1.255513655 |
| --- | --- | --- |

442

443	**Fig 12. Drilling tools from Shubayqa 6.**

444

# 445 Discussion: Craft specialisation and the use of stone beads

446 **in the Pre-Pottery Neolithic**
447 The identification of craft specialisation in the archaeological record has widely been regarded as a
448 challenging task and depends on how analysts define this phenomenon. A universal definition is,
449 however, problematic since craft specialisation is also dependent on the specific economic context and
450 mode of production. We found Wright and Garrard’s [11] criteria, reformulated after Costin [7] for the
451 identification of artisanal activity in the archaeological record, a convenient starting point. According to
452 this, workshops can be inferred if:
453 1. “Artefacts are differentially distributed among production units (households, communities, regions)
454 2. There is a high density of craft production debris relative to some other generally used item
455 3. There are high ratios of unfinished goods to finished goods.” [11]
456 This archaeological definition eschews a more specific economic definition and instead focuses on
457 empirical data, precisely, on what can be discerned from archaeological contexts.
458 What can be gathered from the evidence presented is that the stone bead assemblage from Shubayqa 6
459 stands out amongst other PPNA assemblages in the Levant, because of the distance between the site and
460 the raw material sources, and the overall size of the assemblage combined with evidence for on-site
461 production. Following the above criteria suggested by Wright and Garrard [11] Shubayqa 6 can be
462 considered as a workshop, as these beads were arguably produced to be distributed outside the
463 settlement. This is indicated by the high density of waste production material, and the high ratio of
464 It provides evidence for an at present unique early workshop

unfinished material to finished products.

465 site, as there is no similar example from this period in Southwest Asia regarding quality, quantity and
466 the techno-economic investments. To our knowledge, the only PPNA stone bead assemblage that has
467 also revealed thousands of beads derives from Körtik Tepe in southeast Turkey [42,61]. However, these
468 beads showed evidence of use and were exclusively found in burial contexts, nonetheless attesting to the
469 importance of stone ornaments and their large-scale production in this period. In the mountains of Bal’as in
470 central Syria, PPNA sites have revealed phosphates-based greenstone raw materials, unfinished and
471 finished beads and pendants along with drilling tool [61,62]. Beadmaking activities took place at these
472 sites. The size of the assemblages of the Bal’as region is however quite small in comparison to Shubayqa
473 6, and the production aims seem to be focused on barrel and double-perforated long beads.
474 The detailed technological examination of roughouts and finished beads from Shubayqa 6 suggests a highly
475 standardised *chaîne opératoire*, in which the bead makers at Shubayqa 6 focused their attention on the usage
476 of greenstone raw material for disc bead production. Since the nearest raw material sources are located over
477 60 km away it is clear that Shubayqa 6 bead makers went to considerable lengths to obtain this specific raw
478 material. Stone with a reddish hue was likewise frequently used at Shubayqa 6 that possibly originate from
479 the limestone steppe zone around the Azraq Oasis, where greenstone sources are also situated. These
480 materials were likely obtained either directly from outcrops as part of seasonal procurement trips to the
481 source locations in the Azraq or Wadi Jilat area or through exchange with groups resident in these areas.
482 The presence of Dabba Marble obtained from the Wadi Jilat was confirmed by our preliminary sourcing
483 study discussed above. The XRF study also indicated that the Wadi Jilat was not the only source of stone
484 raw materials for bead making: at least three groups of carbonate greenstones were distinguished, suggesting
485 significant variability and hinting at multiple sources of origin. These other, rarer types of stone were more
486 likely obtained as part of regional exchanges. The presence of marine shells from the Mediterranean and
487 the Red Sea (Alarashi, ongoing analyses), as well as obsidian from Cappadocia [62], indicates the implication
488 of Shubayqa 6 in a complex multi-directional network of circulation.
489 The efforts invested into procuring greenstone raw material from these great distances suggests that these
490 types of raw material had a particular value or meaning that would explain why it was the preferred raw
491 material over other types of stone that were more accessible, such as sandstone and silicified mudstone.
492 This is further exemplified by the instances of reuse of broken roughouts, which suggest that greenstone
493 was considered precious enough to conserve or was hard to come by. At the beginning of the 10th
494 millennium BC, greenstone beads became popular amongst PPNA communities in the Levant (33,34).
495 Thus, it seems that they played a significant role in PPNA societies. At Shubayqa 6 the distance to the raw
496 material sources may have further increased their value. Traveling ~60 km in the challenging terrain of the
497 basalt desert to obtain raw materials for bead production, either through collection at source or over shorter
498 distances through exchange, was not necessarily a straightforward or risk-free endeavour. In many societies
499 access to non-local raw materials helped increase the perceived value of products and access to such sources
500 was often controlled or regulated in specific ways [7,63]. Spielmann [63] has argued that procurement of
501 difficult to access raw materials having particular social value is possibly an impetus towards craft
502 specialisation, and the finds of almost whole raw material nodules at Shubayqa 6 suggests that artisans
503 directly procured at least some of the greenstone for bead manufacture. This procurement of specific types
504 of stones that were, due to their softness and general appearance, considered particularly suitable for the
505 production of beads we see as indicative of specialisation at this site.
506 Once the raw material had been procured it was transformed into beads following a standardised
507 manufacturing sequence that involved flaking, abrasion, perforation and polishing. All these stages were
508 present in the archaeological record of Shubayqa 6 in the form of roughouts and were further attested by
509 the presence of large amounts of nodules, debitage, and the high number of lithic perforators. Based on
510 the reconstructed *chaîne opératoire* it has furthermore been possible to discern some of the technological
511 strategies employed by the bead makers at Shubayqa 6 to accelerate and optimise the manufacturing process.
512 This includes the observations of biconical perforations to avoid risks of breakage, the use of rectangular
513 roughouts to more easily centre the drilling, as well as the polishing of the almost finished beads *en masse*.
514 Thus, bead makers at Shubayqa 6 had attained a high level of skill and were able to accurately judge the
515 qualities of the stone they worked. Combined with the scale and regularity of this workshop across the
516 occupational sequence this provides further evidence for specialised production that was well established
517 and also propagated (taught and learned) through time.
518 Having presented the *châine opératoire* we will now turn our attention to the organisation of this workshop,
519 which we consider to have consisted of part-time specialists. Archaeologists investigating the emergence of
520 craft specialisation have traditionally leaned towards identifying it with the appearance of full-time
521 specialists engaged in highly sophisticated craft activity, such as metalworking [64]. These activities were
522 linked not just to very specific and detailed technological knowledge, practices, workshops and tools, but
523 also to the production of costly prestige goods for elites. However, there is also acknowledgement of the
524 emergence of specialist production of material culture that can be considered more mundane, such as part-
525 time, household-based specialisation of pottery making or flint knapping [6,10], which occurred prior to
526 the emergence of full-time, highly specialised workers. We should not dismiss the apparent skills, knowledge
527 and role these craft workers had, as this level of specialisation was probably much more common in most
528 premodern societies. *Ad hoc*, temporary specialisation is for example evident in many small-scale Palaeolithic
529 and hunting and gathering groups at certain times, and is not usually or necessarily tied to clear social
530 differentiations or inequality [65–68].
531 While we have argued that there is evidence for specialised bead production at Shubayqa 6, and indeed
532 inter-generational transmission of specialist knowledge, we cannot be certain what role or status specialist
533 bead makers amongst the inhabitants of Shubayqa 6 had. When we look at the broader picture of the PPNA
534 in the Levant, we can nevertheless draw on evidence from other sites to discuss what role such early craft
535 specialists may have occupied within PPNA societies. A recurring definition of specialists are craft workers
536 who spend all or most of their time producing a specific type of object. However, this premise can be
537 challenged, as it raises the question of how small-scale PPNA societies could have supported such
538 individuals. Although some sites have produced evidence for dedicated food storage facilities [46] these are
539 neither common nor particularly large, to enable the support of many full-time dedicated specialists.
540 Shubayqa 6 has to date not produced any evidence for storage facilities either. How then can we reconcile
541 the existence of specialist production with the lack of significant surplus food production? The most
542 probable interpretation is that specialist production was carried out by those members of a group that were
543 unable to participate in food procurement activities in a meaningful way. Alternatively, there may also have
544 been a clearer division of tasks within a group, with one segment procuring food and the other focusing on
545 other activities. Finally, it is also conceivable that food procurement simply did not occupy that much of
546 the day-to-day activities and that bead making took place at times when other work was not carried out, as
547 for example, Peschaux et al. demonstrated in the Paris Basin during the Upper Magdalenian period, where
548 bead-making appeared to be seasonally organised [69]. Although we cannot resolve these issues at present,
549 it seems likely that at least at Shubayqa 6 bead making, where it seems to have been a well-developed craft,
550 was only ever carried out by a small and specific group of people. The key point here, however, is that the
551 production of food surplus does not appear to be a requirement for craft specialisation.
552 A further question we wish to briefly reflect on relates to the products of this specialist production: the
553 greenstone beads. What was the incentive for certain individuals to produce disc greenstone beads on a
554 mass scale? One common reason for specialisation to emerge is that there is a demand for certain items as
555 part of trade and exchange networks [7,43]. Given their presence at virtually every site, greenstone beads
556 were clearly in demand with the beginning of the PPNA, which led to the inhabitants of Shubayqa 6 to
557 import raw materials from over 60 km away. Thus, early Neolithic societies appear to have attached some
558 value and meaning to greenstone and the beads made from them. The underrepresentation of finished
559 versus unfinished beads at Shubayqa 6 suggests that beads were not just produced for local use or
560 consumption, but probably for exchange. It is unclear what may have been exchanged for these beads, and
561 it is difficult to say whether this was necessarily a purely economic exchange of goods, or whether beads
562 were exchanged as part of other forms of social interaction (e.g. in gift exchanges, as part of matrimonial
563 exchanges or other purposes). However, it is clear that the inhabitants of Shubayqa 6 made a high socio-
564 economic investment into the manufacture of these beads.
565 The discussion on the usage and meaning of greenstone beads in early Neolithic societies usually centre on
566 these beads being seen as artefacts that have symbolic or ritual meaning, as adornment in the negotiation
567 and establishment of social identities and as circulated trade goods [11,57]. According to Bar-Yosef Mayer
568 & Porat [34], for example, greenstone beads were utilised as charms or amulets protecting the wearer from
569 misfortune and enhancing fertility. While many of these uses and meanings are plausible, we contribute to
570 this discussion with an economic perspective, to arrive at a fuller picture on the use of these beads.
571 We suggest that greenstone beads, as well as beads made from other materials, circulated as an important
572 means of exchange among early Neolithic societies in the Levant. We base this argument on the following
573 observations: disc beads were common and present at most PPNA and PPNB sites throughout the region;
574 the raw material was not easily obtained everywhere, making the raw material rare; and, for reasons that
575 elude us for now, greenstone and similarly colourful stones carried symbolic meaning that made them
576 desired items. Production of greenstone disc beads, as well as other beads, was a time-consuming endeavour
577 that required socio-economic investment. This specialist production would have heightened the value of
578 the final product. Beads occur at sites close to and far from raw material sources and were not produced
579 massively at all sites. This suggests that they were a frequent medium of exchange between groups
580 occupying different localities in the landscape.
581 There is ample ethnographic and archaeological evidence for the use of shell and other beads as a type of
582 currency in societies around the world that may be used to understand the situation we seem to have in the
583 10th – 8th millennium BCE in southwest Asia [70, 71]. What further underlines this comparison is that in
584 these different historical cases beads are considered to have both commercial/symbolic and functional
585 value, as they can also be utilised as amulets or adornments. Importantly, economic anthropologists do not
586 identify the presence of currencies with methods of payment, but foremost as a means of gift exchange,
587 value measurement or as accounting units. Among this the exchange of standardized objects as a means to
588 facilitate exchange is the most important, something which the disc beads could have fulfilled. Regional
589 interaction and exchange have widely been seen as an important aspect of early Neolithic societies in the
590 Levant and southwest Asia generally [72-77], and likely played a crucial role in the emergence of plant
591 cultivation and animal herding practices. Such interaction networks required widely accepted forms and
592 means of exchange and we would argue that stone beads were used in these social contexts. It can even be
593 considered that they were introduced into a much earlier system that was based on marine shell beads,
594 which existed since the Late Epipalaeolithic [78].

595

# 596 Conclusions

597 The orthodox assumption about the emergence of craft specialisation in the human past has associated the
598 appearance of craft workers with the emergence of urban, hierarchical societies, which produced sufficient
599 surplus to enable artisans to specialise in different acts of production. Although it is also acknowledged that
600 a certain level of specialisation is common to all societies, including mobile hunter-gatherers and
601 pastoralists, specialisation has widely been seen as emerging from the kind of social and economic relations
602 existing in early complex societies. In southwest Asia, incipient craft specialisation, including bead
603 manufacture, has at times been put forward for LPPNB societies. This development has been associated
604 with food surplus, the emergence of nuclear households, population growth, as well as emergent social
605 inequality. The evidence from Shubayqa 6 suggests that specialised craft production has its root in a
606 different economic and social setting that does not correspond to standard evolutionary models. Although
607 associated with early plant cultivation, PPNA communities likely produced little significant food surplus,
608 relying instead on a mixed subsistence strategy that involved gathering, cultivating and hunting. These
609 communities were furthermore largely autonomous, with little evidence of social inequality or
610 stratification.
611 An increasingly complex stone bead production evolved throughout the Neolithic Period in Southwest
612 Asia, which in the southern Levant led to the widespread exchange of greenstone beads. An early
613 production centre for these can be found at the site of Shubayqa 6, which has revealed substantial evidence
614 of selective use of imported raw material, a standardised production sequence, and an unevenly distributed
615 ratio of roughouts to finished beads, which all hint towards nascent craft specialisation in stone
616 bead production as early as the PPNA. It provides a case against archaeological and anthropological
617 narratives that place the emergence of craft specialisation in the contexts of early complex societies with
618 food surplus production and increasing social stratification. What initiated the intensified production of
619 greenstone beads, and why these were so widely utilised remains open for discussion. However, we
620 argue that the starting point for understanding this issue lies in the broader social context of their use and
621 their possible role in the establishment and maintenance of early interregional exchange networks.
622 **Acknowledgements**
623 The research reported in this paper was made possible thanks to funding provided from the Independent
624 Research Fund Denmark (Grant # 4001-00068 & #8018-00133B), the Danish Institute in Damascus and
625 the H.P Hjerl Mindefondent for Dansk Palæstinaforskning. We express our sincere gratitude to the
626 Department of Antiquities of Jordan for permission to conduct fieldwork at Shubayqa 6. We are
627 grateful to our local host community in al-Safawi, and especially Mr Ali Shreitir for help in the field. We
628 would like to thank Alexis Pantos for the photographs reproduced in Figures 2-4, 10 & 12, and Lisa
629 Yeomans for the site plan reproduced in Figure 2. We would also like to thank the two anonymous
630 reviewers for their comments. 631

632	**References**

| 633 | 1. | Childe VG. Social Evolution. Cleveland: Meridian Books; 1952. |
| --- | --- | --- |
| 634 | 2. | Childe VG. What happened in history. Harmondsworth, Middlesex: Penguin Books; |
| 635 | 1960. |  |

636 3.	Brumfiel EM, Earle TK. Specialization, Exchange, and Complex Societies: An
637 Introduction. In: Specialization, exchange and complex societies. Cambridge: Cambridge
638 university press; 2008. p. 1–9. (New directions in archaeology).
639 4.	Clark J, Parry W. Craft specialization and cultural complexity. In: Research in
640 economic anthropology 12. 1990. p. 289–346.
641 5.	Stein GJ. Heterogeneity, power, and political economy: Some current research issues
642 in the archaeology of Old World complex societies. J Archaeol Res. 1998 Mar;6(1):1–44.
643 6.	Kerner S. Craft Specialisation and its Relation with Social Organisation in the Late
644 6th to Early 4th Millennium BCE of the Southern Levant. Paléorient. 2010;36(1):179–98.
645 7.	Costin C. Craft Specialization: Issues in Defining, Documenting, and Explaining the
646 Organization of Production. Archaeol Method Theory. 1991;3:1–56.
647 8.	Dobres MA, Hoffman CR. Social agency and the dynamics of prehistoric technology.
648 J Archaeol Method Theory. 1994 Sep;1(3):211–58.
649 9.	Patterson TC. Craft specialization, the reorganization of production relations and state
650 formation. J Soc Archaeol. 2005 Oct;5(3):307–37.
651 10.	Thomas J. Approaching Specialisation: Craft Production in Late Neolithic/Copper
652 Age Iberia. Pap Inst Archaeol. 2009 Oct 13;19(0):67–84.
653 11.	Wright K, Garrard A. Social identities and the expansion of stone bead-making in
654 Neolithic Western Asia: new evidence from Jordan. Antiquity. 2003 Jun;77(296):267–84.
655 12.	Rosen SA. Lithics after the Stone Age: a handbook of stone tools from the Levant.
656 Walnut Creek, CA: AltaMira Press; 1997. 184 p.
657 13.	Goring-Morris N, Belfer-Cohen A. Highlighting the PPNB in the Southern Levant.
658 NEO-LITHICS. 2020;20:3–22.
659 14.	Barzilai O. Social complexity in the southern Levantine PPNB as reflected through
660 lithic studies: the bidirectional blade industries. Oxford: Archaeopress; 2010. 194 p. (BAR
661 international series).
662 15.	Schick T. Nahal Hemar Cave: Cordage, Basketry and Fabrics. Atiqot. 1988;18:31–43.
663 16.	Goren Y, Goring-Morris AN. Early pyrotechnology in the Near East: Experimental
664 lime-plaster production at the Pre-Pottery Neolithic B site of Kfar HaHoresh, Israel.
665 Geoarchaeology. 2008 Nov;23(6):779–98.
666 17.	Abbes F. Les outillages néolithiques en Syrie du Nord. Méthode de débitage et
667 gestion laminaire durant le PPNB. Oxford: Archaeopress; 2003. (BAR International
668 Series).
669 18.	Kirkbride D. Five Seasons at the Pre-Pottery Neolithic Village of Beidha In Jordan.
670 Palest Explor Q. 1966 Jan;98(1):8–72.
671 19.	Alarashi H. Butterfly Beads in the Neolithic Near East: Evolution, Technology and
672 Socio-cultural Implications. Camb Archaeol J. 2016 Aug;26(3):493–512.
673 20.	Alarashi H. La parure épipaléolithique et néolithique de la Syrie (12e au 7e millénaire
674 avant J.-C.) : Techniques et usages, échanges et identités. Volume 2. 2018 [cited 2023 Feb
675 24]; Available from: <http://rgdoi.net/10.13140/RG.2.2.30494.38726>
676 21.	Alarashi H. New insights into the technological management of the Neolithic cowrie
677 beads in the Levant: An experimental and traceological approach. In: Beyond Use-Wear
678 Traces Going from tools to people by means of archaeological wear and residue analyses,
679 2021, p171-183, Sidestone press. Sidestone press; 2021. p. 171–83.
680 22.	Bar-Yosef Mayer DE, Groman-Yaroslavski I, Bar-Yosef O, Hershkovitz I, Kampen-
681 Hasday A, Vandermeersch B, et al. On holes and strings: Earliest displays of human
682 adornment in the Middle Palaeolithic. Petraglia MD, editor. PLOS ONE. 2020 Jul
683 8;15(7):e0234924.
684 23.	Bar-Yosef Mayer DE, Vandermeersch B, Bar-Yosef O. Shells and ochre in Middle
685 Paleolithic Qafzeh Cave, Israel: indications for modern behavior. J Hum Evol. 2009
686 Mar;56(3):307–14.
687 24.	Maher LA, Richter T, Stock JT. The Pre-Natufian Epipaleolithic: Long-term
688 Behavioral Trends in the Levant. Evol Anthropol Issues News Rev. 2012 Mar;21(2):69–81.
689 25.	Bar-Yosef Mayer DE. Nassarius shells: Preferred beads of the Palaeolithic. Quat Int.
690 2015;390:79–84.
691 26.	Stiner M. Prey choice, site occupation intensity & economic diversity in the Middle –
692 early Upper Palaeolithic at the Üçağizli Caves, Turkey. Farming. 2009 Jan;2009(3):1–20.
693 27.	Stiner MC, Kuhn SL, Güleç E. Early Upper Paleolithic shell beads at Üçağızlı Cave I
694 (Turkey): Technology and the socioeconomic context of ornament life-histories. J Hum Evol.
695 2013 May;64(5):380–98.
696 28.	Le Dosseur G, Maréchal C. Bone ornamental elements and decorated objects of the
697 Natufian from Mallaha. In: Bar-Yosef O, Valla FR, editors. Natufian Foragers in the Levant:
698 Terminal Pleistocene Social Changes in Western Asia [Internet]. 1st ed. Berghahn Books;
699 2013 [cited 2023 Feb 25]. p. 293–311. Available from:
700 <http://www.jstor.org/stable/10.2307/j.ctv8bt33h>
701 29.	Maréchal C. Eléments de parure de la fin du Natoufien : Mallaha niveau I, Jayroud 1,
702 Jayroud 3, Jayroud 9, Abu Hureyra et Mureybet IA. In: Bar-Yosef O, editor. The Natufian
703 culture in the Levant. Ann Arbor, Mich: International Monographs in Prehistory; 1991. p.
704 120–1. (International monographs in prehistory Archaeological series).
705 30.	Wyllie C, Hole F. Wyllie C, Hole F (2012) Personal adornment in the Epi-Paleolithic
706 of the Levant. In: Matthews R, Curtis J (eds) Proceedings of the 7th International Congress
707 on the Archaeology of the Ancient Near East, 12 April – 16 April 2010, the British Museum
708 and UCL, London. Wiesbaden: Harrassowitz Verlag, 707–717. In: Proceedings of the 7th
709 International Congress on the Archaeology of the Ancient Near East: 12 April - 16 April
710 2010, the British Museum and UCL, London. Wiesbaden: Harrassowitz; 2012. p. 707–17.
711 31.	Torres HR, Groman-Yaroslavski I, Weinstein-Evron M, Yeshurun R. A micro-wear
712 analysis of Natufian gazelle phalanx beads from el-Wad Terrace, Mount Carmel, Israel. J
713 Archaeol Sci Rep. 2020 Jun;31:102304.
714 32.	Kurzawska A, Bar-Yosef Mayer DE, Mienis HK. Scaphopod Shells in the Natufian
715 Culture. In: Bar-Yosef O, Valla FR, editors. Natufian Foragers in the Levant: Terminal
716 Pleistocene Social Changes in Western Asia [Internet]. 1st ed. Berghahn Books; 2013 [cited
717 2023 Feb 25]. p. 611–21. Available from: <http://www.jstor.org/stable/10.2307/j.ctv8bt33h>
718 33.	Bar-Yosef Mayer DE. Towards a typology of stone beads in the Neolithic Levant. J
719 Field Archaeol. 2013 May;38(2):129–42.
720 34.	Bar-Yosef Mayer DE, Porat N. Green stone beads at the dawn of agriculture. Proc
721 Natl Acad Sci. 2008 Jun 24;105(25):8548–51.
722 35.	Alarashi H. PPNA Stone Grooved Pendants from the Middle Euphrates Valley:
723 Markers of Cultural Identity? NEO-LITHICS. 2016;16:20–9.
724 36.	Maréchal C, Alarashi H. Les éléments de parure de Mureybet. In: Ibáñez, J.J. (Ed.),
725 Le site néolithique de Tell Mureybet (Syrie du Nord). In: Ibáñez Estévez JJ, Cauvin J,
726 Maison de l’Orient et de la Méditerranée Jean Pouilloux (Lyon, France), editors. Le site
727 néolithique de Tell Mureybet (Syrie du Nord): en hommage à Jacques Cauvin. Oxford:
728 Archaeopress; 2008. p. 575–617. (BAR international series).
729 37.	Benz M, Gresky J, Štefanisko D, Alarashi H, Knipper C, Purschwitz C, et al. Burying
730 power: New insights into incipient leadership in the Late Pre-Pottery Neolithic from an
731 outstanding burial at Baʻja, southern Jordan. Biehl PF, editor. PLOS ONE. 2019 Aug
732 28;14(8):e0221171.
733 38.	Benz M, Gresky, J., Alarashi, H. Similar but different: displaying social roles of
734 subadults in burials from the Late Pre-Pottery Neolithic site of Ba`ja, Southern Jordan. In:
735 Alarashi H, Dessì RM, editors. L’art du paraître: apparences de l’humain, de la Préhistoire à
736 nos jours = The art of human appearance: from prehistory to the present day: actes des
737 rencontres, 22-24 octobre 2019. Nice: Éditions APDCA; 2020. p. 93–107.
738 39.	Groman-Yaroslavski I, Bar-Yosef Mayer DE. Lapidary technology revealed by
739 functional analysis of carnelian beads from the early Neolithic site of Nahal Hemar Cave,
740 southern Levant. J Archaeol Sci. 2015 Jun;58:77–88.
741 40.	Raad DR, Makarewicz CA. Application of XRD and digital optical microscopy to
742 investigate lapidary technologies in Pre-Pottery Neolithic societies. J Archaeol Sci Rep. 2019
743 Feb;23:731–45.
744 41.	Wright KI, Critchley P, Garrard A, Baird D, Bains R, Groom S. Stone Bead
745 Technologies and Early Craft Specialization: Insights from Two Neolithic Sites in Eastern
746 Jordan. Levant. 2008 Nov;40(2):131–65.
747 42.	Baysal E. A tale of two assemblages: Early Neolithic manufacture and use of beads in
748 the Konya plain. Anatol Stud. 2013 Dec;63:1–15.
749 43.	Baysal E. Will the real specialist please stand up? Characterising early craft
750 specialisation, a comparative approach for Neolithic Anatolia. Doc Praehist. 2013 Dec
751 8;40:233–46.
752 44.	Twiss K. The Complexities of Home Cooking. In: Pollock S, editor. Between the
753 Feasts and Daily Meals. Berlin Studies of the Ancient World 30. 2015 [cited 2023 Feb 24];
754 Available from: <http://edition-topoi.org/articles/details/the-complexities-of-home-cooking>
755 45.	Kuijt I. Demography and Storage Systems During the Southern Levantine Neolithic
756 Demographic Transition. In: Bocquet-Appel JP, Bar-Yosef O, editors. The Neolithic
757 Demographic Transition and its Consequences [Internet]. Dordrecht: Springer Netherlands;
758 2008 [cited 2023 Feb 24]. p. 287–313. Available from: <http://link.springer.com/10.1007/978->
759 1-4020-8539-0_11
760 46.	Kuijt I, Finlayson B. Evidence for food storage and predomestication granaries 11,000
761 years ago in the Jordan Valley. Proc Natl Acad Sci. 2009 Jul 7;106(27):10966–70.
762 47.	Ibáñez-Estévez JJ, Anderson PC, Arranz-Otaegui A, González-Urquijo JE, Jörgensen-
763 Lindahl A, Mazzucco N, et al. Sickle gloss texture analysis elucidates long-term change in
764 plant harvesting during the transition to agriculture. J Archaeol Sci. 2021 Dec;136:105502.
765 48.	Pedersen PN. The Groundstone Assemblages of Shubayqa 1 and 6, eastern Jordan -
766 Technological Choices, gestures and processing strategies of late hunter-gatherers in the Qa’
767 Shubayqa. In: Pedersen PN, Jörgensen-Lindahl A, Sørensen M, Richter T, editors.
768 Proceedings of the 3rd Meeting of the Association of ground stone tools research. Oxford
769 (GB): Archaeopress publishing Ltd; 2021. p. 18–42. (Access archaeology).
770 49.	Bronk Ramsey C. Bayesian Analysis of Radiocarbon Dates. Radiocarbon.
771 2009;51(1):337–60.
772 50.	Reimer PJ, Austin WEN, Bard E, Bayliss A, Blackwell PG, Bronk Ramsey C, et al.
773 The IntCal20 Northern Hemisphere Radiocarbon Age Calibration Curve (0–55 cal kBP).
774 Radiocarbon. 2020 Aug;62(4):725–57.
775 51.	Finlayson B, Warren GM. Changing natures: hunter-gatherers, first farmers and the
776 modern world. London: Duckworth; 2010. 144 p. (Duckworth debates in archaeology).
777 52.	Karlin C, Bodu P, Pelegrin J. Processus techniques et chaines opératoires. Comment
778 les préhistoriens s’approprient un concept élaboré par les ethnologues. In: Balfet H, editor.
779 Observer l’action technique. Paris: Editions du CNRS; 1991. p. 101–17.
780 53.	Pelegrin J, Karlin C, Bodu P. Pelegrin, J., Karlin, C., Bodu, P. 1988	“Chaînes
781 opératoires” : un outil pour le préhistorien. In: Technologie préhistorique, Notes et
782 monographies techniques du CRA 25. CNRS Editions, Paris, pp. 55–62. In: Journee d
783 ’Etudes Technologiques en Prehistoire. Paris: CNRS Editions; p. 55–62.
784 54.	Alarashi H. La parure épipaléolithique et néolithique de la Syrie (12e au 7e millénaire
785 avant J.-C.) : Techniques et usages, échanges et identités. Volume 1. [Lyon]: Université
786 Lumière-Lyon; 2014.
787 55.	Barthélémy de Saizieu B, Bouquillon A. Steatite working at Mehrgarh during the
788 neolithic and chalcolithic periods: quantitative distribution, characterization of material and
789 manufacturing processes. In: European Association of South Asian Archaeologists, Parpola
790 A, Koskikallio P, editors. South Asian Archaeology, 1993: proceedings of the Twelfth
791 International Conference of the European Association of South Asian Archaeologists held in
792 Helsinki University, 5-9 July 1993. Helsinki: Suomalainen Tiedeakatemia; 1994. p. 47–59.
793 (Suomalaisen Tiedeakatemian toimituksia. Sarja B, Annales academiae scientiarum fennicae.
794 Ser B).
795 56.	Beck HC. I.— *Classification and Nomenclature of Beads and Pendants*.
796 Archaeologia. 1928;77:1–76.
797 57.	Bains RK. The Social Significance of Neolithic Stone Bead Technologies at
798 Çatalhöyük. Institute of Archaeology, University College London; 2012.
799 58.	Hauptmann A. 'Greenstones’ from Basta. Their mineralogical composition and
800 possible provenance. In: Nissen H, Muheisen M, Gebel HGK, editors. Basta 1: The human
801 ecology. Berlin: Ex Oriente; 2004. p. 169–76. (Monograph of the Faculty of Archaeology and
802 Anthropology).
803 59.	Critchley P. The Stone Beads. In: Finlayson B, Mithen SJ, Council for British
804 Research in the Levant, editors. The early prehistory of Wadi Faynan, Southern Jordan:
805 archaeological survey of Wadis Faynan, Ghuwayr and al-Bustan and evaluation of the pre-
806 pottery neolithic A site of WF16. Oxford: Oxbow; 2007. p. 356–61. (Levant supplementary
807 series).
808 60.	Thuesen MB, Kinzel M. Stone Beads from Shkārat Msaied. NEO-LITHICS.
809 2018;18(1):3–7.
810 61.	Özkaya V, Coşkun A. Körtik Tepe. In: Başgelen N, Ozdoğan M, Kuniholm PI,
811 editors. The Neolithic in Turkey: new excavations & new research. 3rd ed. Galatasaray,
812 İstanbul: Archaeology & Art Publications; 2011. p. 89–127.
813 62.	Campbell S, Healey E. Manchester Obsidian Laboratory, Lab Report 130. University
814 of Manchester; 2020.
815 63.	Spielmann KA. Feasting, Craft Specialization, and the Ritual Mode of Production in
816 Small-Scale Societies. Am Anthropol. 2002 Mar;104(1):195–207.
817 64.	Gero J, Scattolin M. Beyond Complementarity and Hierarchy: New Definitions for
818 Archaeological Gender Relations. In: Nelson SM, Rosen-Ayalon M, editors. In pursuit of
819 gender: worldwide archaeological approaches. Walnut Creek, CA: AltaMira Press; 2002. p.
820 155–71. (Gender and archaeology series).
821 65.	Conkey MW. Ritual Communication, Social Elaboration, and the Variable
822 Trajectories of Paleolithic Material Culture. In: Prehistoric Hunters-Gatherers [Internet].
823 Elsevier; 1985 [cited 2023 Feb 24]. p. 299–323. Available from:
824 https://linkinghub.elsevier.com/retrieve/pii/B9780125647502500160
825 66.	Heckel CE. Reconsidering production organization in the Early Upper Palaeolithic:
826 The case for specialized production of Aurignacian beads. Quat Int. 2018 Oct;491:11–20.
827 67.	Sassaman KE. Crafting Cultural Identity in Hunter-Gatherer Economies. Archeol Pap
828 Am Anthropol Assoc. 2008 Jun 28;8(1):93–107.
829 68.	Tibbett K. Community specialisation, standardisation and exchange in a hunter-
830 gatherer society: a case study from Kalkadoon country, northwest Queensland, Australia
831 [Internet]. James Cook University; 2005 [cited 2023 Feb 24]. Available from:
832 https://researchonline.jcu.edu.au/1255/
833 69.	Peschaux C, Bignon-Lau O, Debout G, Bodu P. Magdalenian « beadwork time » in
834 the Paris basin (France) : correlation between personal ornaments and the function of
835 archaeological sites. In: Mayer DEBY, Bonsall C, Choyke AM, editors. Not Just for Show:
836 The Archaeology of Beads, Beadwork, and Personal Ornaments [Internet]. Havertown:
837 Oxbow Books; 2017 [cited 2023 May 31]. p. 19–38. Available from:
838 <http://www.jstor.org/stable/10.2307/j.ctv13pk763>
839 70.	Gamble LH. The origin and use of shell bead money in California. J Anthropol
840 Archaeol. 2020 Dec;60:101237.
841 71.	Trubitt MBD. The Production and Exchange of Marine Shell Prestige Goods. J
842 Archaeol Res. 2003;11(3):243–77.
843 72.	Alarashi H, Chambrade ML. Outils géographiques appliqués à l’étude de la
844 provenance des matériaux utilisés pour la parure néolithique L’exemple du site de Mureybet.
845 In: Alarashi H, Chambrade ML, Gondet S, Jouvenel A, Sauvage C, Tronchère H, editors.
846 Regards croisés sur l’étude archéologique des paysages anciens Nouvelles recherches dans le
847 Bassin méditerranéen, en Asie centrale et au Proche et au Moyen-Orient. Lyon: Maison de
848 l’Orient; 2010. p. 95–106.
849 73.	Asouti E. Beyond the Pre-Pottery Neolithic B interaction sphere. J World Prehistory.
850 2006 Dec;20(2–4):87–126.
851 74.	Bar-Yosef O, Belfer-Cohen A. The Levantine “PPNB” Interaction Sphere. In:
852 Hershkovitz I, editor. People and Culture in Change. 1989. p. 59–72. (BAR International
853 Series; vol. 1).
854 75.	Borrell F, Molist M. Social Interaction at the End of the Pre-Pottery Neolithic B: an
855 Inter-site Analysis in the Euphrates Valley. Camb Archaeol J. 2014 Jun;24(2):215–32.
856 76.	Ibáñez JJ, Ortega D, Campos D, Khalidi L, Méndez V. Testing complex networks of
857 interaction at the onset of the Near Eastern Neolithic using modelling of obsidian exchange. J
858 R Soc Interface. 2015 Jun;12(107):20150210.
859 77.	Watkins T. Supra-Regional Networks in the Neolithic of Southwest Asia. J World
860 Prehistory. 2008 Jun;21(2):139–71.
861 78.	Richter T, Garrard AN, Allock S, Maher LA. Interaction before Agriculture:

| 862 | Exchanging Material and Sharing Knowledge in the Final Pleistocene Levant. Camb |
| --- | --- |
| 863 | Archaeol J. 2011 Feb;21(1):95–114. |
| 864 |  |
| 865 | **Supporting information** |
| 866 | **S1 Appendix. Shubayqa 6 XRF Data.** |
| 867 | **S2 Appendix. Greenstone Bead Experiment Report.** |
| 868 | **S3 Appendix. Bead analysis data** |

869 **S2 Fig 1. The piece of green Dabba Marble from Wadi Jilat that was used for the experiment.**
870 **S2 Fig 2. Abrading the greenstone fragment into a rectangular preform on non-vesicular basalt.**
871 **S2 Fig 3. Cutting the greenstone with a flint blade**
872 **S2 Fig 4. Drilling of a rectangular roughout.**
873 **S2 Fig 5. The beads strung together with sinew.**
874 **S2 Fig 6. En masse polishing.**
875 **S2 Table 1. Time measures for all the finished beads according to the different manufacturing**
876 **stages.**
877 **S2 Table 2. Overview of the failed roughouts and the cause of breakage.**
878 **S2 Table 3. The drilling techniques used for the finished beads.**

879
